# Analysis of chromatin accessibility uncovers TEAD1 as a regulator of migration in human glioblastoma

**DOI:** 10.1038/s41467-018-06258-2

**Published:** 2018-10-01

**Authors:** Jessica Tome-Garcia, Parsa Erfani, German Nudelman, Alexander M. Tsankov, Igor Katsyv, Rut Tejero, Bin Zhang, Martin Walsh, Roland H. Friedel, Elena Zaslavsky, Nadejda M. Tsankova

**Affiliations:** 1 Department of Pathology, Icahn School of Medicine at Mount Sinai, New York, NY 10029 USA; 2 Department of Neuroscience and The Friedman Brain Institute, Icahn School of Medicine at Mount Sinai, New York, NY 10029 USA; 3 Department of Neurology, Icahn School of Medicine at Mount Sinai, New York, NY 10029 USA; 4 Broad Institute of MIT and Harvard, Cambridge, MA 02142 USA; 5 Department of Genetics and Genomic Sciences and Icahn Institute for Genomics and Multiscale Biology, Icahn School of Medicine at Mount Sinai, New York, NY 10029 USA; 6 Department of Pharmacological Sciences, Center for RNA Biology and Medicine, New York, NY 10029 USA

## Abstract

The intrinsic drivers of migration in glioblastoma (GBM) are poorly understood. To better capture the native molecular imprint of GBM and its developmental context, here we isolate human stem cell populations from GBM (GSC) and germinal matrix tissues and map their chromatin accessibility via ATAC-seq. We uncover two distinct regulatory GSC signatures, a developmentally shared/proliferative and a tumor-specific/migratory one in which TEAD1/4 motifs are uniquely overrepresented. Using ChIP-PCR, we validate TEAD1 trans occupancy at accessibility sites within *AQP4*, *EGFR*, and *CDH4*. To further characterize TEAD’s functional role in GBM, we knockout TEAD1 or TEAD4 in patient-derived GBM lines using CRISPR-Cas9. TEAD1 ablation robustly diminishes migration, both in vitro and in vivo, and alters migratory and EMT transcriptome signatures with consistent downregulation of its target *AQP4*. TEAD1 overexpression restores *AQP4* expression, and both TEAD1 and AQP4 overexpression rescue migratory deficits in TEAD1-knockout cells, implicating a direct regulatory role for TEAD1–AQP4 in GBM migration.

## Abstract (web summary)

The intrinsic drivers of glioblastoma (GBM) migration are still poorly understood. Here the authors purify GBM stem cells (GSCs) from patients and profile chromatin accessibility in these cells, identifying TEAD1 as a regulator of migration in human glioblastoma.

## Introduction

Glioblastoma (GBM) is the most common primary brain tumor in adults, carrying dismal prognosis despite aggressive treatment. The diffusely infiltrative nature of tumor growth in GBM greatly confounds surgical therapy, as infiltrative cells inevitably extend beyond the resection margin. Moreover, glioma cells away from the tumor’s contrast-enhancing core respond poorly to chemotherapy, and have been implicated in tumor recurrence^1–3^. Given the unique microenvironment and transcriptional signatures of tumor cells at the infiltrative edge vs. those at the tumor core^4,5^, the two populations are likely regulated by distinct molecular pathways.

Epigenetics is critical for allowing plasticity during normal stem-cell development and differentiation^6,7^ as well as for the maintenance of an aberrant cancer stem-cell state^8–10^. In GBM, chromatin remodeling supports the re-emergence of developmental programs in glioma stem cells (GSCs), leading to progressive tumor growth^8,10–15^. The regulatory promoter/enhancer regions at key developmentally driven oncogenes, such as the epidermal growth factor receptor (*EGFR*)^16^, are maintained in a poised or open chromatin state, enabling their accessibility for transcriptional dysregulation in GBM^3^. In this context, several transcriptional pathways and transcription factor (TF) drivers of cell proliferation have been uncovered in cultured GSC/tumor-initiating cells^8,10–12,15^, and in GSC populations prospectively isolated from human samples^17^.

Many studies have also identified TF regulators of migration and invasion in cultured GSCs or patient-derived xenografts (PDX) mouse glioma models^18^, including SMADs acting downstream of the TGFβ signaling pathway^19^, HIF1/2-alpha^20,21^, ZEB1^22,23^, STAT3^24^, C/EBPβ^25^, TWIST1^26^, SNAIs^27^, and Id2^28^. However, the extent to which these TFs modulate intrinsic cell migration in patients with GBM has been more difficult to discern, due to the complex adaptations of infiltrative tumor cells within their microenvironment for motility, adhesion, hypoxia, metabolism, and immune response^1^. To this end, transcriptome analysis of primary GBM samples defined STAT3 and C/EBPβ as master regulators of mesenchymal transformation^29^, a behavior typically associated with increased tumor infiltration and GBM recurrence; and ZEB1 was found to be significantly overexpressed in infiltrative vs. non-infiltrative glioma lesions^22^. Analysis of TFs in human GSC populations, directly isolated from their niche, may uncover additional intrinsic regulators of migration without the caveats of cell culture and intermixed normal brain parenchyma.

Here, we utilize a recently developed protocol for the prospective isolation of GSCs and neural stem/progenitor cells (NSPCs) directly from human GBM and germinal matrix (GM) tissues^17,30^ in order to study the intrinsic transcriptional regulators of tumorigenicity in a comparable developmental context. By contrasting chromatin accessibility in developing and neoplastic stem cell populations, we define transcriptionally accessible regions unique to GSCs that specifically relate to cell migration and which are highly enriched for TEAD1/4 motifs. We subsequently provide functional validation for the role of TEAD1 in GBM migration, both in vitro and in vivo, implicate several oncogenic binding targets of TEAD1, and show that TEAD1 directly regulates AQP4 expression to promote cell migration.

## Results

### Distinct chromatin accessibility regions in uncultured GSCs

**Fig. 1:**
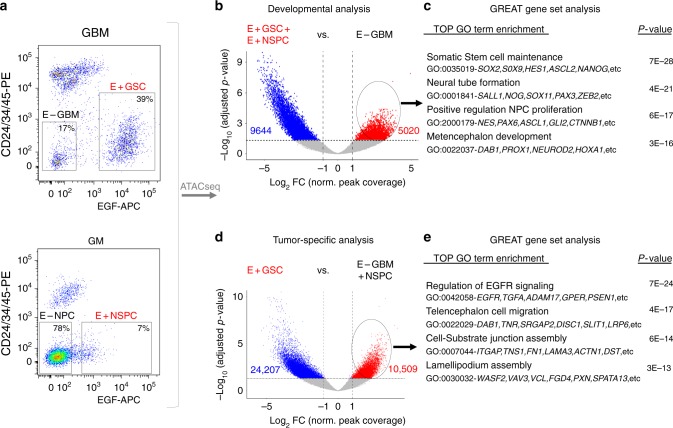
Chromatin accessibility analysis in acutely isolated human GSCs and NSPCs. **a** FACS plot showing isolation strategy of GBM and neural germinal matrix (GM) populations used for ATAC-seq preparation. See also Supplementary Fig. 1. **b** Differential ATAC-seq peak analysis between the union of E+GSC and E+NSPCs (*n* = 7) and E–GBM (*n* = 3) defines 5020 significantly enriched, developmentally shared peaks in E+GSCs. **c** Functional enrichment analysis of developmentally shared E+GSC peaks using GREAT. Illustrated are top significantly enriched gene ontology (GO) terms and examples of the corresponding annotated genes, which relate to stem-cell maintenance and proliferation. **d** Differential ATAC-seq peak analysis between E+GSC (*n* = 4) and the union of E–GBM and NSPCs (*n* = 9) defines 10,509 significantly enriched peaks unique to tumor E+GSCs. **e** Functional enrichment analysis of GSC tumor-specific peaks using GREAT. Illustrated are top significantly enriched GO terms and examples of the corresponding annotated genes, which relate cell migration, cell-substrate assembly, and EGFR signaling

Recent studies have highlighted striking similarities in chromatin plasticity between development and cancer, which support progressive GSC growth^8,10,16,17^. Yet, the two processes are clearly different: neural development is coordinated while glial tumors overtake the brain through uncontrolled growth and infiltrative spread. To better understand the similarities and differences between neural progenitors and GSCs, we performed comparative analysis of chromatin accessibility in acutely isolated human NSPC and GSC populations, using the assay for transposase-accessible chromatin with sequencing (ATAC-seq). ATAC-seq enables broad assessment of open chromatin using the ability of the Tn5 transposase to bind and tagment accessible nucleosome-free regions, accurately identifying regulatory regions for transcription in small populations of cells^31^. Human NSPCs were isolated from autopsy GM dissections, and GSCs from isocitrate dehydrogenase (IDH)-wildtype GBM surgical specimens, by fluorescence-activated cell sorting (FACS), based on their EGF ligand-binding ability (Fig. 1a, Supplementary Fig. 1); this purification strategy prospectively captures all sphere-forming and tumor-initiating EGF-bound cells in uncultured conditions^17^. ATAC-seq data were obtained from both EGF-bound (E+) and E− fractions extracted from GBM and GM tissues. We first aligned reads and defined all open chromatin regions (peaks) in our samples. The data showed comparable reads per peak frequency across samples and open chromatin distribution along the genome, with expected enrichment at promoters^31,32^ (Supplementary Fig. 2). We then performed a series of differential accessibility analyses. To specifically assess the developmental contributions of chromatin plasticity in GSCs, we compared the union of E+GSCs and E+NSPCs stem cell enriched populations to E−GBM sorted cells, the latter lacking stem cell properties in vitro or tumor initiation in vivo^17^. We obtained a defined set of 5020 differential peaks in both GSCs and NSPCs (Fig. 1b, Supplementary Data 1). Using the Genomic Regions Enrichment of Annotations Tool (GREAT)^33^, we annotated these peaks with their closest genes and then performed a functional enrichment analysis. The developmentally shared GSC/NSPC peak-associated genes related to biological processes of stem cell maintenance and proliferation (Fig. 1c), paralleling previously observed transcriptome similarities in these populations^17^. We then focused our analysis on the differences between GSC and NSPC, in an effort to define regulatory elements that are unique to the tumor stem cell phenotype. To do so, we performed differential accessibility analysis in E+GSCs vs. all other cell populations (the union of NSPCs and E−GBMs). This analysis defined a set of 10,509 peaks specifically enriched in tumoral GSCs, corresponding to 4015 protein-coding genes (Fig. 1d, Supplementary Data 1), which showed remarkable association with cell migration, motility, and extracellular matrix (ECM) processes (Fig. 1e). Similar functional gene set associations were seen when comparing E+GSC to NSPC transcriptome (Supplementary Fig. 3a–b). Thus, by uniquely contrasting chromatin accessibility in GBM to early germinal growth, we were able to distinguish developmental and tumor-specific chromatin states within GSCs in uncultured conditions, and to define a regulatory signature that relates to GSC migration.

### TEAD motifs are prioritized at GSC-specific open chromatin

**Fig. 2:**
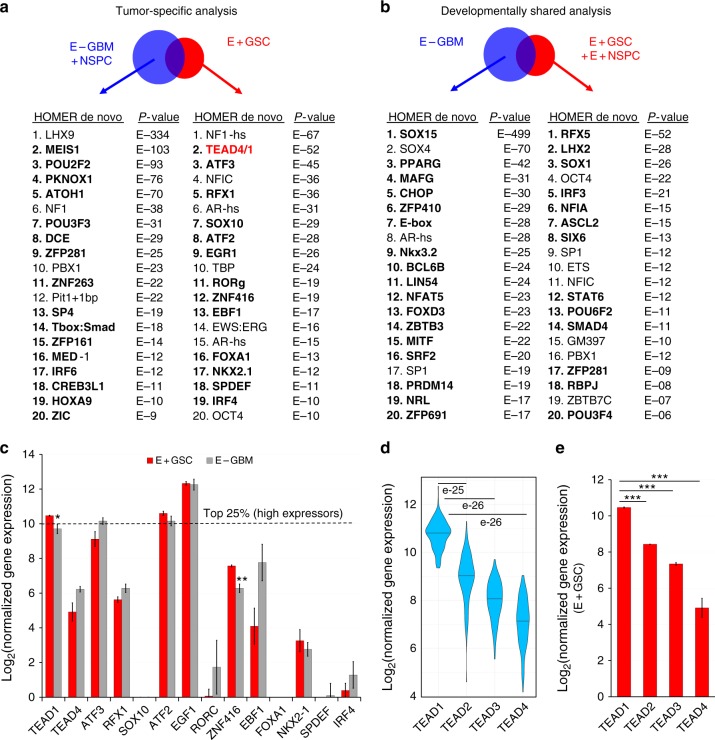
TEAD is the top selectively enriched motif at GSC-specific open chromatin and *TEAD1* is its most highly expressed family member across GBMs **a**, **b** Homer de novo motif discovery outlines the 20 most highly enriched TF motifs at chromatin accessibility regions defined by the GSC tumor-specific (**a**) and developmentally shared (**b**) differential ATAC-seq peak analyses (motifs in bold show selective enrichment in only one peak set). The TEAD motif (with highest scores for TEAD4 and TEAD1) is the top, selectively enriched motif within differential GSC tumor-specific peaks (in red). See also Supplementary Data 1. **c** Bar graph of rld-normalized gene expression values for all significantly and uniquely enriched GSC tumor-specific TF motifs, generated from parallel RNA-seq data in E+GSC and E−GBM populations. *TEAD1* is the only highly expressed gene (top 25th percentile), which is differentially overexpressed in E+GSCs (*p = 0.0496 for TEAD1, **p = 0.006 for ZNF416, *n* = 3. Bars represent mean ± SEM). **d** Violin plot of *TEAD1-4* expression in TCGA GBM RNA-seqV2 data (*n* = 150) shows that *TEAD1* is the most highly expressed TEAD family member, followed by *TEAD2*, *TEAD3*, and *TEAD4*. Expression shown is log2(normalized counts + 1), normalized as detailed in methods. *p*-Values from one-sided Wilcoxon matched pairs test. Bar represents median value. **e** Bar graph of rld-normalized gene expression values for *TEAD1-4* derived from RNA-seq E + GSC data (***p < 0.001; *n* = 3. Bars represent mean ± SEM)

Analysis of chromatin accessibility via ATAC-seq not only identifies regulatory regions for transcription, but can also infer TF activity within them^31,32^ and has been recently used to identify regulators of neuronal fate and de-differentiation in cultured GBM cells and cortical neural progenitors^12,34,35^. Given this, we set to define TF candidates at accessible chromatin regions in GSCs and GM NSPCs without cell culture exposure. Using the HOMER de novo motif-discovery algorithm, we identified a list of 27 and 26 TF motifs, significantly enriched within GSC tumor-specific or within developmentally shared peak sets, respectively, compared to all-peaks background (Fig. 2a, b, Supplementary Data 1). Of these, we only considered those motifs, which were exclusively enriched in only one of the two peak set analyses (in tumor-specific or in developmentally shared) but not in both (Fig. 2a, b, in bold). For example, NFIC was not considered because it was enriched at #4 in the tumor-specific analysis but also at #11 in the developmentally shared analysis. This highlighted TEAD1/4 as the most highly and uniquely overrepresented motifs in GSC tumor-specific peaks. Other top GSC tumor-specific candidates included ATF3, RFX1, SOX10, ATF2, EGR1, RORg, ZNF416, EBF1, FOXA1, etc. (Fig. 2a, in bold). In contrast, the most highly uniquely overrepresented TF motifs at developmentally shared GSC peaks included RFX5, LHX2, SOX1, IRF3, NFIA, ASCL2, SIX6, STAT6, POU6F2, SMAD4, etc. (Fig. 2b) and PAX6 (Supplementary Data 1). We then considered the gene expression levels of tumor-specific TF candidates, using previously generated RNA-seq data from similar E+/E–GBM cells^17^. Four TFs with enriched tumor-specific motifs showed high gene expression (top 25th percentile) of which only *TEAD1* was differentially overexpressed in E+GSCs (Fig. 2c).

### *TEAD1* is the most highly expressed TEAD member across GBMs

To evaluate the relevance of TEAD1 across GBM subtypes, we analyzed the expression levels of all TEAD family members (1–4) in RNA-seq data obtained from The Cancer Genome Atlas (TCGA) database^36,37^. We found *TEAD1* to be the most highly expressed TEAD family member across 150 primary GBM samples (Fig. 2d), which paralleled expression patterns observed in acutely isolated GSC populations (Fig. 2e). Of note, genes significantly coexpressed with *TEAD1* in TCGA GBM samples were highly enriched for terms related to cell migration and cell adhesion (Supplementary Fig. 3c). At the protein level, we noted expression of TEAD1 but not of other TEAD members in PDX gliomas previously generated from acutely sorted GBM GSCs^17^ (Supplementary Fig. 4). Overall, this analysis prioritized TEAD1 as the most highly and widely expressed TEAD family member across GBM tumors.

### Ablation of TEAD1/4 impairs migration in primary GBM lines

**Fig. 3:**
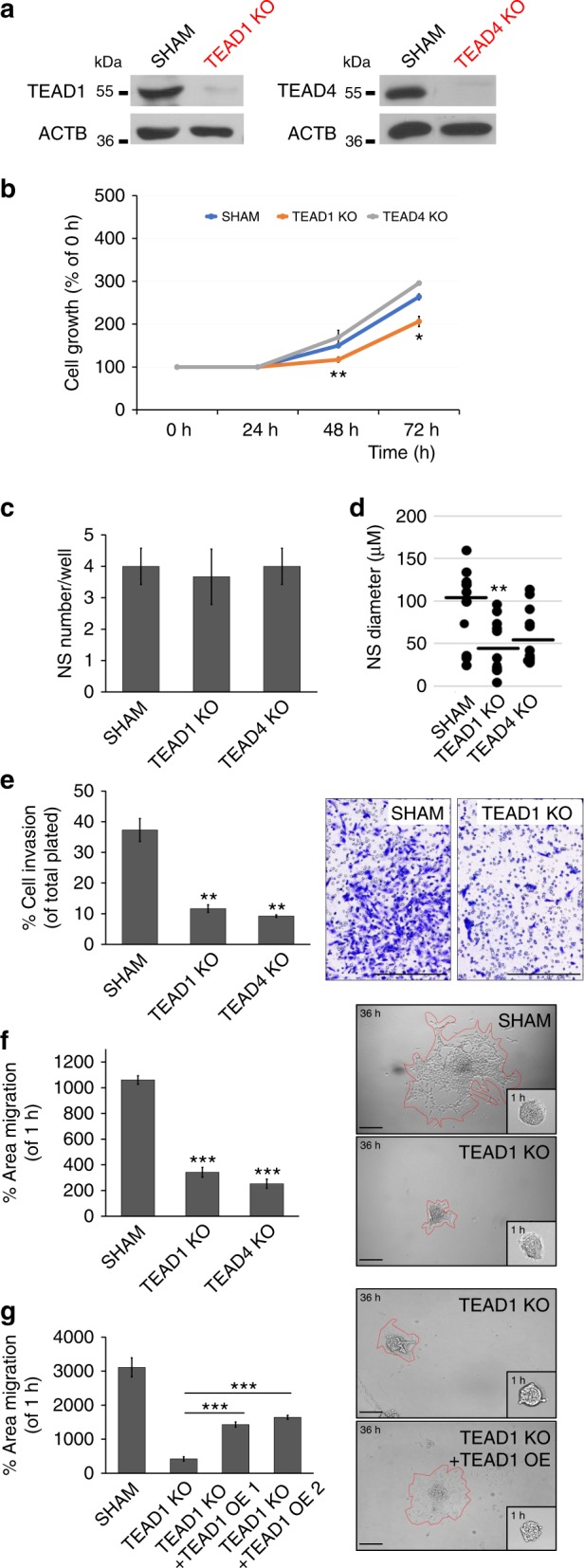
CRISPR-Cas9 ablation of TEAD1/4 inhibits migration in primary GBM cells. **a** Western immunoblot confirms population knockout of TEAD1 and TEAD4 after CRISPR-Cas9-mediated gene ablation. **b** Cell growth analysis reveals significantly decreased proliferation in TEAD1KO cells at 48–72 h, compared to sham (*n* = 3; 48 h: **p = 0.008; 72 h: *p = 0.01. Bars represent mean ± SEM). **c** Neurosphere (NS) assays show no difference in sphere number (day 6; *n* = 3 wells, multiple NS per well. Bars represent mean ± SEM). **d** Neurosphere (NS) assays show decreased sphere size in TEAD1 knockout, compared to sham. (day6; *n* = 3 wells, multiple NS per well; TEAD1KO: **p = 0.002. Dots represent individual NS and lines delineate mean). **e** Transwell invasion assays show decreased percent cell invasion in TEAD1KO and TEAD4KO cells, compared to sham (24 h; *n* = 3 wells; TEAD1KO: **p = 0.002; TEAD4KO: **p = 0.001. Bars represent mean ± SEM). On right, representative images of transwell invasion chamber membranes are shown. **f**, **g** Spheroid migration assays show decreased area of confluent cell migration (dispersion) at 36 h in TEAD1KO and TEAD4KO cells, compared to sham (**f**, PDL substrate), with partial rescue of migratory deficits in TEAD1KO cells after TEAD1 overexpression (OE) (**g**, laminin + PDL substrate) (*n* = 3 wells, with multiple NS per well; TEAD1KO: ***p = 0.0001; TEAD4KO: ***p = 0.00007; TEAD1OE 1: ***p = 0.00058; TEAD1OE 2 (1/10 dilution of TEAD1OE 1): ***p = 0.00015. Bars represent mean ± SEM). On right, migration area marked by red dash line in representative spheroids is shown. All experiments in **a**–**g** are performed on G-13063 cells. Scale bars = 75 μM. See also Supplementary Fig. 5

TEAD2/4 activity has been recently implicated in GBM motility and mesenchymal transformation^38^. However, the specific role of TEAD1, the most highly expressed TEAD member in GBM, remains undefined. To validate experimentally the role of TEAD1 in GBM migration, we generated stable population knockout of TEAD1, and its better studied paralog TEAD4, in patient-derived, low-passaged GBM cells, by using CRISPR-Cas9 genome editing to introduce loss-of-function mutations (Fig. 3a, Supplementary Fig. 5a–b). As a negative control, we generated a sham CRISPR-Cas9 knockout targeting the non-human GFP gene.

First, we assessed whether knockout (KO) of TEAD1 or TEAD4 affected GBM growth in vitro. Cell proliferation in serum-free conditions was evident in all groups at ~36 h after laminin attachment, and TEAD1KO cells were less proliferative compared to sham at 48–72 h after attachment (Fig. 3b). Under low attachment conditions, in the absence of a substrate, sham and TEAD1/4KO cells formed a similar number of spheres (Fig. 3c), but interestingly, TEAD1KO spheres were significantly smaller in size (Fig. 3d) and showed reduced frequency for self-renewal in extreme limited dilution assays (ELDA) (Supplementary Fig. 5c). We then assessed whether elimination of TEAD1 or TEAD4 affects cell migration at early time points, 24 and 36 h, when proliferation differences are not yet evident.

Genetic ablation of TEAD1 or TEAD4 resulted in substantial reduction of cell migration, compared to sham treatment, in two independent in vitro migration assays, the transwell invasion assay (Fig. 3e) and the spheroid dispersion assay (Fig. 3f, Supplementary Movies 1 and 2). To confirm the observed phenotype across additional GBM subtypes, we generated CRISPR-Cas9 population TEAD1 and TEAD4 knockouts in three additional primary GBM cell lines, G-13306, G-12746, and G-16302 (Supplementary Fig. 5d), representing various GBM molecular subtypes (Supplementary Table 1). We observed migratory deficits in these additional lines after knockout of TEAD1 or TEAD4 (Supplementary Fig. 5e–f). Finally, to test if TEAD1 activity is regulating cell migration directly, we overexpressed TEAD1 in TEAD1KO cells and assessed their spheroid migration behavior. TEAD1 overexpression rescued migration deficits in TEAD1KO spheroids by ~50% (Fig. 3g, Supplementary Movie 3), strengthening further the regulatory role of TEAD1 for GBM migration in vitro.

### TEAD1-knockout GBM cells show infiltrative deficits in vivo

**Fig. 4:**
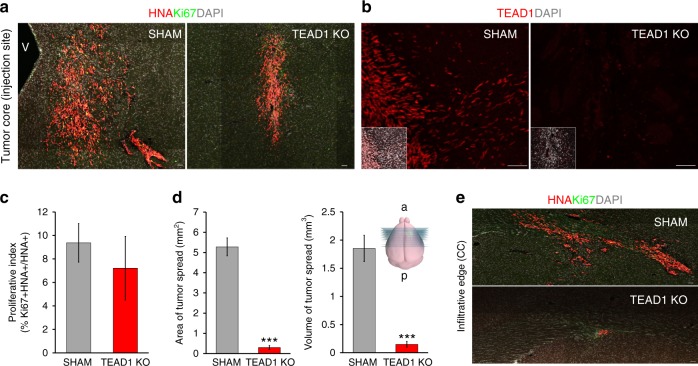
In vivo infiltration is highly impaired in TEAD1KO GBM xenografts. **a** Representative immunofluorescent histology of tumor engraftment and core growth near the injection site in sham and TEAD1KO cells, 3.5 months after orthotopic xenotransplantation. **b** Representative immunofluorescence images of TEAD1 expression, confirming the stable loss of TEAD1 in TEAD1KO xenografts. Insets represent the entire TEAD1 immunofluorescence image with DAPI co-labeling. **c** Quantification of cell proliferation in sham and TEAD1KO cells at 3.5 months post xenotransplantation (*n* = 3 animals per condition; Ki67+ cells counted out of all HNA+ cells in 11 serial histological sections. Bars represent mean ± SEM). **d** Quantification of migratory tumor spread in sham and TEAD1KO cells at 3.5 months post xenotransplantation (*n* = 3 animals per condition; ***p = 0.0003 for both. Area = sum of areas with tumor spread in 11 serial coronal sections. Volume = average area × the distance between the first and the last serial section examined. Bars represent mean ± SEM). Schematic of the analyzed mouse brain serial coronal sections is depicted on the right. **e** Representative example of infiltrative tumor spread along the corpus callosum in sham and TEAD1KO cells at 3.5 months post xenotransplantation. HNA: human nuclear antigen, CC: corpus callosum, v: ventricle, a anterior, p: posterior. Scale bar = 50 μM

To confirm whether the anti-migratory deficits observed in cell culture are recapitulated in vivo, we performed orthotopic xenotransplantation using sham and TEAD1KO primary GBM cells, choosing the TEAD1KO line with the most robust in vitro anti-migratory effect. Histological analysis at 3.5 months after transplantation, a relatively early time point in PDX formation, confirmed tumor engraftment in both sham and TEAD1KO cells (Fig. 4a) as well as the stable loss of TEAD1 protein in TEAD1KO cells (Fig. 4b). Sham and TEAD1KO cells also showed similar cell proliferation index (Fig. 4c). In contrast, TEAD1KO cells displayed significant deficits in two and three-dimensional infiltrative tumor spread across the mouse brain, compared to sham (Fig. 4d). While sham cells had infiltrated extensively into the striatum, away from the tumor injection site, and across the corpus callosum, TEAD1KO cells were found primarily around the site of injection with only occasional infiltrative cells counted throughout the rest of the brain (11 serial coronal sections examined entirely) (Fig. 4d, e). This provided additional evidence for the role of TEAD1 in GBM migration and prompted further studies to define its downstream pro-migratory targets.

### Validation of TEAD1 binding targets

The transcriptional targets of TEADs have been predominantly studied in tumors outside of the brain, where they have been implicated in tumor invasion, metastasis, and epithelial-to-mesenchymal transition (EMT)^39–44^. To embark on the complex characterization of TEAD1 targets in GBM migration, tumors known for their heterogeneous nature and unique diffusely infiltrative growth, we undertook two initial approaches: (1) analysis of putative TEAD1 targets ex vivo using parallel ATAC-seq and RNA-seq datasets generated from acutely isolated GSCs^17^ and (2) analysis of putative TEAD1 targets in vitro using TEAD1KO vs. Sham RNA-seq data generated from four different GBM cell lines.

**Fig. 5:**
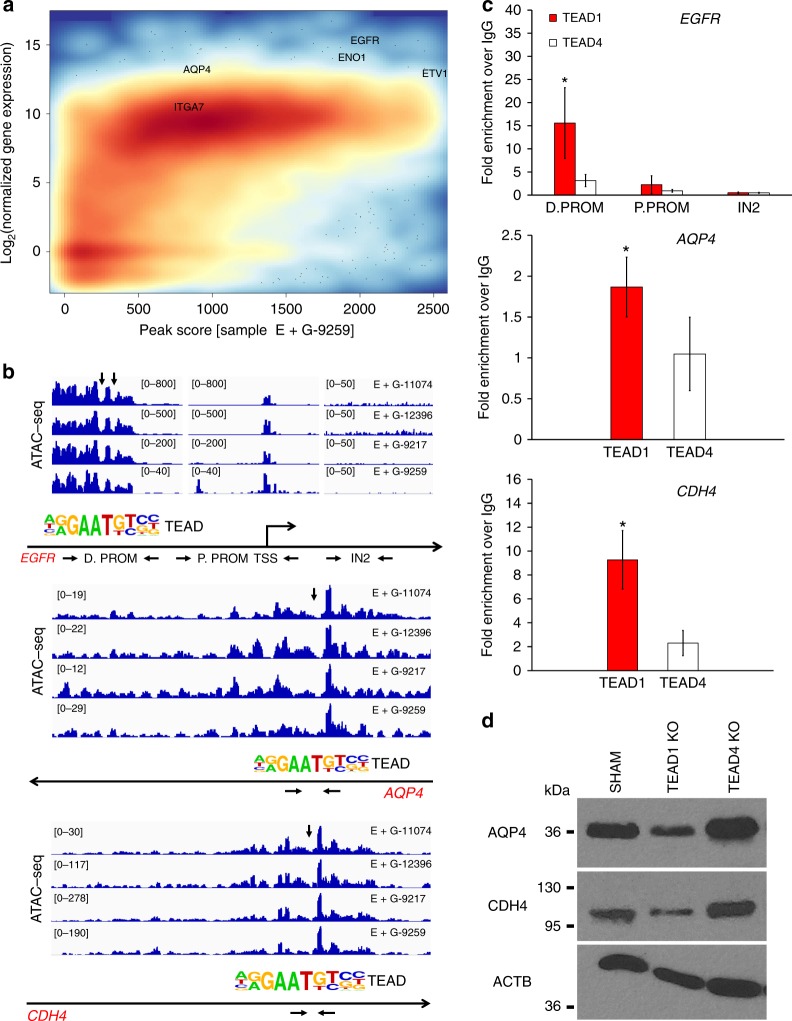
Validation of TEAD1-binding targets by chromatin immunoprecipitation. **a** Density plot of correspondence analysis between chromatin accessibility and gene expression. Plotted on the *y*-axis is the average rld-normalized gene expression for each gene from all RNA-seq E+GSC sample data. Plotted on the *x*-axis is the highest ATAC-seq peak associated with the proximal promoter [−5 kb, +3 kb] of the same gene. Color intensity indicates density of the gene population, with red representing higher densities and blue representing lower densities. Strong correspondence is observed between open chromatin peaks and a moderate/high level of gene expression in all E+GSC samples (plot for one representative sample shown here). Several putative TEAD1-target genes of interest are indicated on the plot. **b** IGV plot of ATAC-seq piled reads at *EGFR*, *AQP4*, and *CDH4* in four different acutely sorted E+GSCs (D.PROM distal promoter of EGFR, peak180759: chr7:55,000,372–55,001,595; P.PROM proximal promoter to TSS of EGFR, peak180777: chr7:55,085,981–55,088,747). For this IGV representation, reads are centered on the cut site of the Tn5 enzyme, correcting for the 9 bp occupancy of Tn5, and presumed footprints/peak troughs corresponding to TEAD motifs are delineated by downward arrow. **c** Chromatin immunoprecipitation (ChIP-PCR) in GBM tissues. Significant enrichment of TEAD1 (but not TEAD4) over IgG is seen specifically at differential open chromatin peaks with associated TEAD1 motif within the upstream promoter of *EGFR* (D. PROM, chr7:55001141 motif start site) (*n* = 6, *p = 0.011), at *CDH4* (chr20:60011935 motif start site) (*n* = 6, *p = 0.027), and at the proximal promoter of *AQP4* (chr18:24444251 motif start site) (*n* = 6, *p = 0.029). Significant binding above background is not observed at *EGFR* P.PROM or at chromatin inaccessible regions (IN2 = EGFR intron 2). Enrichment is expressed as fold increase over IgG, after normalization with 10% input, using 2^–ΔΔCt analysis accounting for primer efficiency: (*E*^IA−*S*)_sample_/(*E*^IA−*S*)_IgG_. *E*: primer efficiency; IA: 10% Input-Adjusted Ct; *S*: sample Ct. Bars represent mean ± SEM. **d** Western immunoblot illustrates decreased expression of AQP4 and CDH4 in TEAD1KO but not in TEAD4KO G-13063 GBM cells

For the first (ex vivo) analysis, we made the assumption that transcription and TF-chromatin accessibility are functionally linked, which was supported by the strong correspondence between moderate-high levels of gene expression and the presence of open chromatin within gene promoters in parallel RNA-seq and ATAC-seq E+GSC datasets (Fig. 5a). The most highly differentially expressed genes (log2(fold change) >3; E+GSCs vs. E-GBM+NSPCs analysis) with enriched TEAD-accessible chromatin peaks corresponded to WNT, Cadherin, EGFR, and metabolism-related signaling pathways, many of them previously implicated in tumor invasion^18^, and included *EGFR*, *CDH4*, *TNC*, *AQP4*, *ETV1*, *ITGA7*, *ENO1*, *PCDHGC5*, *NRCAM*, *PTPRZ1*, *ACSBG1*, *MEOX2*, *OSMR*, and *ACSS3* (Supplementary Fig. 6a–b). Most of these genes were significantly coexpressed with *TEAD1* in the TCGA GBM RNA-seq data analysis (Supplementary Data 2). We also considered the number of TEAD-associated peaks present within a gene with linked GSC overexpression and their TEAD motif scores. The highest number of peaks/gene, by far, was at *EGFR* (18), and five or more peaks were noted at *NRCAM* (9), the cadherins *CDH2/4/11* (8), *ETV1* (7), *AQP4* (6), and *TNC* (5) (Supplementary Data 1). This analysis provided several potential (*cis*) binding targets for TEAD1 in acutely isolated GSC populations.

**Fig. 6:**
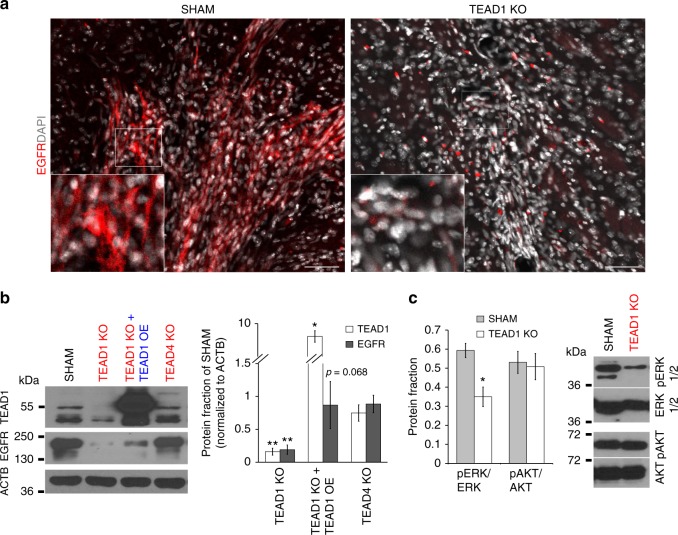
TEAD1 regulates expression of EGFR. **a** Immunofluorescence image depicts observed expression of EGFR in sham and TEAD1-knockout G-13063 xenografts 3.5 months post transplantation. Scale bar = 50 μM. **b** Western immunoblot depicts marked downregulation of EGFR in vitro after knockout of TEAD1, but not TEAD4, in G-13063 cells, which is partially restored after TEAD1 overexpression for 48 h. On right is a bar graph quantification of immunoblots from three independent experiments (*n* = 3; TEAD1KO vs. TEAD4KO: **p = 0.004 and **p = 0.003 for TEAD1 and EGFR, respectively; TEAD1KO + TEAD1OE vs. TEAD1KO: *p = 0.01 for TEAD1; *p* = 0.068 for EGFR in one tail *t*-test analysis. Bars represent mean ± SEM). **c** Western immunoblot depicts downregulation of pERK/ERK but not pAKT/AKT in TEAD1KO cells, compared to sham (*n* = 3 cell lines; pERK/ERK: *p = 0.029; bars represent mean ± SEM). On right are shown representative immunoblot images from one cell line

To validate whether the above TEAD1-associated loci are binding targets of TEAD1 in trans, we performed chromatin immunoprecipitation (ChIP-PCR) in human GBM tumors of different subtypes (Supplementary Table 1), using previously validated TEAD1 and TEAD4 antibodies^38,39,45^. We assessed several regulatory regions for which we had previously demonstrated chromatin accessibility peak enrichment associated with robust TEAD motif score (Fig. 5b). As an internal negative control, we also measured TEAD1 binding at a region lacking open chromatin^16^ and TEAD accessibility (*EGFR IN2*). ChIP-PCR detected significant enrichment of TEAD1 at TF-accessible regions associated with *EGFR*, *AQP4*, and *CDH4* (Fig. 5c) but not with *TNC*, *ETV1*, *NRCAM*, *CDH11*, or *CDH2* (Supplementary Fig. 6c). Accordingly, expression of the TEAD1-binding targets AQP4, CDH4, and EGFR was downregulated in TEAD1KO (but not in TEAD4KO) cells (Figs. 5d and 6a, b).

### TEAD1 is a direct but transient regulator of EGFR in vitro

Given the known role of EGFR signaling in GBM tumorigenesis^3^, we focused further on the relationship between TEAD1 and EGFR activity. In vivo, we found co-expression of TEAD1 with EGFR in PDX GBM tumors (Supplementary Fig. 4a) and observed decreased expression of EGFR in TEAD1KO xenografts (Fig. 6a). In vitro, we detected decreased expression of EGFR after TEAD1 knockout, with a trend for partial rescue after TEAD1 overexpression (Fig. 6b). We extended our analysis to downstream EGFR signaling components and found consistent downregulation of pERK but not pAKT in TEAD1KO cells, compared to sham (Fig. 6c). The downregulation of EGFR in TEAD1KO cells, however, appeared to be transient. After several months of cell culture with growth factor supplementation, TEAD1KO cells regained EGFR expression but continued to show deficits in spheroid migration (Supplementary Fig. 6e, Supplementary Data 3). This indicated that TEAD1’s effect on EGFR expression is not directly related to cell migration in vitro and prompted a more global, agnostic search for TEAD1-dependent regulators within a migration-specific framework.

### TEAD1 regulates migration by modulating AQP4 expression

**Fig. 7:**
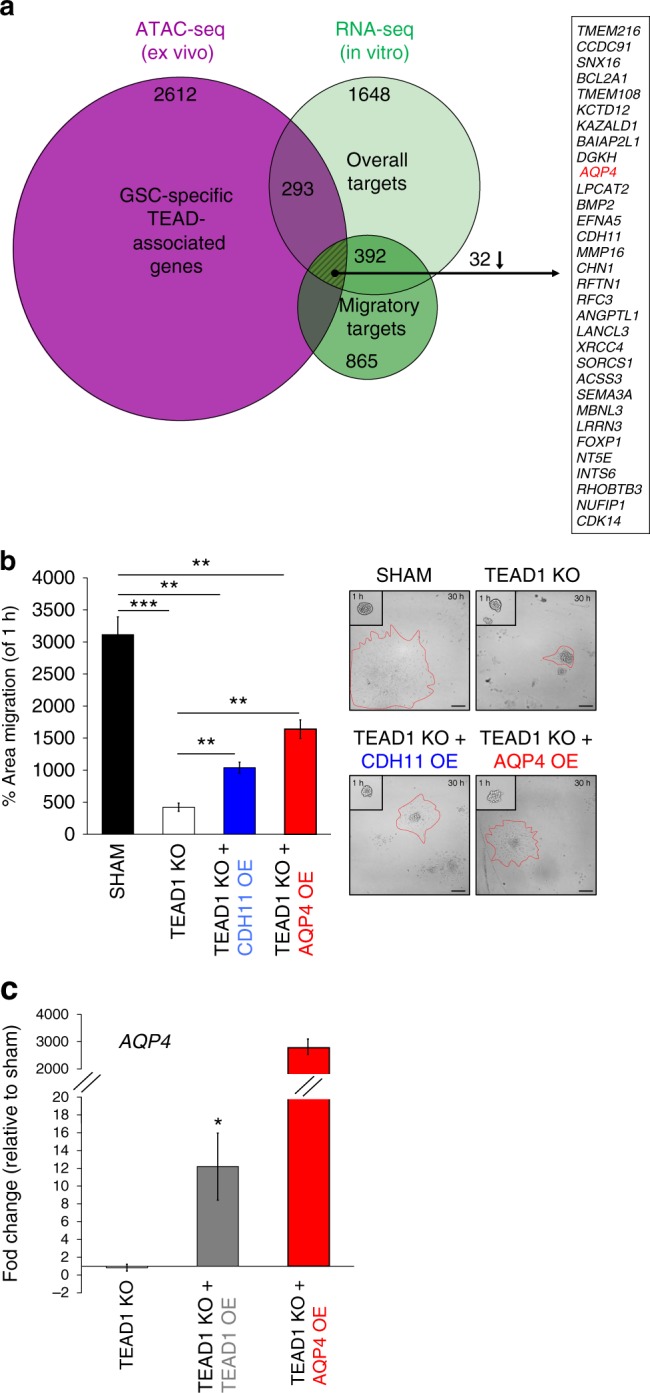
TEAD1 regulates GBM migration by modulating AQP4 expression. **a** Venn diagram depicts the intersection of genes with TEAD motifs and genes consistently downregulated in TEAD1KO cell lines and migration-deficient spheroids (striped area). AQP4 is the only one of 32 genes in this intersection found to be a direct TEAD1 binding target in vivo (highlighted in red). ATAC-seq set contains 2612 peak-annotated genes from E+GSC vs. E−GBM+NSPC differential accessibility analysis. Top RNA-seq set (overall targets) contains all 1648 significantly up or downregulated genes from TEAD1KO vs. Sham differential expression analysis in all samples from four different patient-derived GBM lines and three migration experiments (*n* = 7; *p*adj. < 0.05; all log2(fold change) values of HGNC-annotated genes included). Bottom RNA-seq set (migratory targets) contains 865 significantly up or downregulated genes from TEAD1KO vs. Sham G-13063 spheroids from three independent migration experiments (*n* = 3; *p*adj. < 0.05; log2(fold change) >1 or <−1). See also Supplementary Fig. 7. **b** Spheroid migration assay showing significant reversal of cell dispersion deficit at 30 h in TEAD1 knockout cells after overexpression of CDH11 or AQP4 (G-13063 line; laminin + PDL substrate; *n* = 3 wells. TEAD1KO + CDH11OE: **p = 0.004; TEAD1KO + AQP4OE: **p = 0.0015. Bars represent mean ± SEM). On right, migration area marked by red dash line in representative spheroids is shown. Scale bar = 75 μM. **c** Quantification of *AQP4* expression by RT-qPCR. *AQP4* is significantly upregulated in TEAD1KO cells after TEAD1 overexpression (*n* = 6, 4 technical and 2 biological replicates; *p = 0.02 TEAD1KO + TEAD1OE vs. TEAD1KO) and is robustly expressed after exogenous lentivirus overexpression. Bars represent mean ± SEM

To gain further insight into the regulatory targets of TEAD1 in the context of GBM migration, we analyzed RNA-seq data from sham, TEAD1KO, and TEAD4KO samples generated from four different cell lines (“overall target” analysis, Supplementary Fig. 7a) as well as from sham and TEAD1KO spheroids, the latter showing robust anti-migratory behavior (“migratory target” analysis, Supplementary Fig. 7b). Principal component analysis of all samples revealed expected clustering by patient-specific cell line (Supplementary Fig. 7a), as well as strong separation by migration phenotype in TEAD1KO vs. Sham spheroids (Supplementary Fig. 7b). Functional enrichment analysis of differentially downregulated TEAD1KO genes was statistically significant for terms related to cell migration and “EMT” gene set (Supplementary Fig. 7c–e). In contrast, differential analysis of TEAD4KO vs. Sham samples did not reveal a significant number of dysregulated genes (Supplementary Fig. 7c). To narrow down potential TEAD1 migratory targets that are relevant across GBM subtypes, we analyzed the intersection between TEAD1KO-dysregulated genes in the “overall target” and “migratory target” analyses. We found 392 overlapping genes, and out of all downregulated ones, 32 had TEAD-associated chromatin accessibility peaks (Fig. 7a). Again, we noted downregulation of several pro-migratory genes (Supplementary Data 3), including *CDH11*^46^ and *AQP4*^47–49^, the latter also found to be a direct TEAD1 (*trans*)binding target in GBM, in vivo (Fig. 5).

To gain mechanistic insight into how TEAD1 regulates tumor migration, we overexpressed AQP4 and CDH11 in sham and TEAD1KO spheroids and studied their migratory behavior. Overexpression of either AQP4 or CDH11 partially restored confluent migration in TEAD1KO spheroids when grown under laminin plus PDL substrate (Fig. 7b, Supplementary Movies 4 and 5). In the case of AQP4 overexpression, migratory abilities of TEAD1KO cells improved by ~50%, recapitulating the effect seen after TEAD1 overexpression (Fig. 3g, Supplementary Movie 3). The effect of CDH11 overexpression was less pronounced but still significant. Notably, along with promoting spheroid migration, overexpression of TEAD1 also upregulated endogenous expression of *AQP4* (Fig. 7c). In contrast, CDH11 expression was not rescued after TEAD1 overexpression (Supplementary Fig. 6d). Overall, these data provide further evidence that both AQP4 and CDH11 play a role in GBM migration. Importantly, it also demonstrates a direct mechanistic link between TEAD1 and downstream AQP4 expression for promoting migratory properties in primary GBM cells.

## Discussion

Understanding the intrinsic molecular mechanisms that control GBM cell migration may uncover opportunities for more effective therapy aimed at inhibiting infiltration early in the disease course, in order to optimize surgical resection and chemoradiation. An attractive strategy to study the intricate biology of migration is to use epigenetics to define its transcriptional regulators in a system that recapitulates the cell’s native tumor state^3,50^. Here we hypothesized that the analysis of chromatin accessibility in glioma and neural stem cell populations, isolated prospectively from GBM and GM niches without prior culture or genetic manipulations, may capture some of the intrinsic and perhaps distinct TF drivers for cell migration in GSCs.

The recently described chromatin accessibility (ATAC-seq) assay allows for the high-resolution sequencing of TF-accessible chromatin in low number of cells, thus providing an ideal platform to infer TF activity in our rare, fresh tissue-derived human stem cell populations. Importantly, we had previously demonstrated that such FACS-isolated E+NSPC and E+GSC populations capture all sphere-forming cells in vitro, with the capacity for both self-renewal and tri-lineage differentiation, and that E+GSC populations are tumor-initiating in vivo, capable of re-forming GBM-like tumors after orthotopic xenotransplantation^17^. To hone in on the unique regulatory signature of GSCs and to minimize their heterogeneity, we computationally extracted the developmental and non-GSC chromatin contributions from the E+GSC ATAC-seq data, by performing differential peak analysis of E+GSC vs. E−GBM+NSPCs. By contrasting the chromatin landscape in tumor GSCs to that of the highly proliferative early GM progenitors, we were able to expose a unique regulatory signature in GSCs that specifically relates to tumoral cell migration. This analysis allowed us to uncover several enriched GSC-specific TF motifs, some of which had been previously implicated in GBM invasion^51–53^. We focused our downstream experimental validation studies on TEAD1, the most highly and uniquely overrepresented motif candidate within the migratory GSC accessibility signature, whose expression was highest among TEAD family members across the GBM TCGA database, yet its role in GBM tumorigenesis had not been previously established.

The oncogenic activity of TEAD proteins has been predominantly described outside of the CNS and in the context of Hippo pathway signaling, where their interaction with the transcriptional co-activators YAP/TAZ has been implicated in cell growth, invasion, EMT, and metastasis^44^. Well-characterized TEAD targets in non-CNS tumors include *CTGF*, *Cyr61*, *MYC*, *CCNE1*, *AREG*, and *EGFR*^44,54,55^. Our study uncovered that EGFR is also a downstream target of TEAD1 in GBM, likely through the direct binding of TEAD1 to an upstream promoter/enhancer region at *EGFR*. Curiously, in vitro the regulatory effect of TEAD1 on EGFR was transient, and not directly related to migration, while in vivo TEAD1-knockout xenografts showed low levels of EGFR after 3.5 months. The discrepancy may be related to the artificial EGF-driven growth conditions in cell culture and the redundancy of pathways driving EGFR signaling in the context of oncogenic addiction. We cannot exclude compensatory regulation by other TEAD family members as well. Several mechanisms of interaction between EGFR and Hippo pathway effectors YAP/TAZ-TEAD have been recently demonstrated in epithelial tumors^55–58^, some of which have been also implicated in tumor chemoresistance^55,59–61^. Whether EGFR also crosstalks with upstream members of the Hippo family in GBM, as seen with other tumors, remains to be determined in future studies.

Recent studies have implicated TEADs as master regulators of cell invasion in melanoma^42^. Few studies have also begun to investigate YAP-TEAD activity in GBM malignancy^38,62–68^. Specifically, disruption of TEAD2/4 binding to TAZ or shRNA-mediated knockdown of TAZ has been shown to result in deficient GBM cell migration^38^. Our study expands the current literature by also demonstrating a role for TEAD1 in GBM migration, using both in silico and functional analyses, the latter involving stable CRISPR-Cas9-mediated knockout of TEAD1/4 in primary GBM cultures. While both TEAD1- and TEAD4-knockout cells showed migratory deficits in vitro, TEAD1 was the most highly expressed TEAD member across TCGA GBM samples, underscoring its relevance under physiological conditions. Indeed, we found migratory deficits in TEAD1 knockout GBM cells not only in vitro but also in patient-derived xenografts, strengthening the role of this TF for promoting infiltrative spread in human glioblastoma.

To gain further insight into the downstream effectors of TEAD activity in GBM, we characterized extensively TEAD1/4’s transcriptome across four primary GBM lines and in the context of deficient migration. We discovered a defined TEAD1-knockout transcriptome signature related to migration and EMT but did not find consistently dysregulated genes across TEAD4-knockout lines. Given the expected differences between in vivo and in vitro tumor behavior, we did not observe extensive overlap between TEAD targets inferred from ATAC-seq data (in acutely derived cells) and from RNA-seq data (in cultured cells). For instance, while EGFR was a TEAD1-binding target in vivo, its downregulation in TEAD1KO cells was only transient in vitro. Nevertheless, we did observe lasting downregulation of several TEAD-accessible genes previously implicated in glioma migration, consistently across TEAD1-knockout cell lines and spheroids, including *AQP4*^47–49^, *CDH11*^46^, *MMP16*^69^, *SEMA3A*^70^, and *CDK14*^71^. *AQP4* was also found to be a direct TEAD1-binding target in vivo. Importantly, overexpression of *AQP4* (both exogenously and endogenously after TEAD1 overexpression) resulted in nearly 50% rescue of migratory function, implicating AQP4 as one of TEAD1’s direct regulatory targets for migration in the spheroid dispersion model. While CDH11 overexpression also reversed partially migratory deficits in TEAD1-knockout spheroids, its expression was not directly regulated by TEAD1 and we did not detect TEAD1 occupancy in chromatin accessible regions associated with *CDH11*. This attests to the complex regulatory networks that impact migratory biology, which undoubtedly extend beyond TEAD1 and include other regulators of gene expression, such as other TFs, co-factors, long non-coding and micro RNAs, as well as post-translational modifications.

Several studies have previously demonstrated a role for AQP4, a major water channel expressed in CNS astrocytes, in tumor edema and migration^47–49,72–75^, the mechanisms of which are still incompletely understood. AQP4 is highly expressed at the leading edge of tumor cells, polarizing to and increasing the number and size of tumor lamellipodia to ensure water influx during cell movement^76^. AQP4 may also play a role in cytoskeletal organization^77^. Our study points toward a TEAD1-mediated transcriptional regulation of AQP4, a previously unrecognized TEAD1-target interaction, which affects GBM migration in vitro. The exact mechanism by which TEAD1-AQP4 affect tumor migration under physiological conditions remains to be determined. Given the well-known role of YAP-TEAD Hippo effectors in actin polarization and motility^44^, it is tempting to speculate that AQP4’s role in cytoskeletal organization may be through the Hippo-TEAD pathway.

## Methods

### Sample collection and processing

All specimen collection was performed de-identified in accordance with the policies and regulation at the Icahn School of Medicine at Mount Sinai (ISMMS) and its institutional review board. Unfixed tissues from GBM resections and GM postmortem dissections (19–22 weeks of gestation) were dissociated mechanically and enzymatically (papain, 0.3 mg ml^−1^), following published protocols^17,30^. Fluorochrome-conjugated EGF ligand (E) (ThermoFisher, E35351, 5 μg per million alive cells) was used along with PE-CD24/CD34/CD45 antibodies (BD, Biosciences, 560991, 550619, 555483, 1:10) to prospectively FACS-isolate GM and GBM cells with stem cell properties (referred to as E+NSPCs and E+GSCs) while excluding ependymal/endothelial/ inflammatory cells^17,30,78^. DAPI incorporation (4′,6-Diamidino-2-Phenylindole, Dihydrochloride, ThermoFisher, D1306, 1:1000) was used for dead cell exclusion. In GBM, DAPI^low^ gating was used to further enrich for tumor populations. FACS-sorted E+/E− (CD24−/CD34−CD45−) GM and GBM cells were immediately used for downstream ATAC-seq preparation, without freezing.

### Chromatin accessibility data preparation and analysis

ATAC-seq sample and library preparation was performed using the prokaryotic Tn5 transposase system (Nextera DNA library kit, Illumina, FC-121–1030)^31^, using 13–100 K sorted cells starting material (most samples contained 25 K cells), Tn5 incubation time of 50 min, and 9–14 PCR cycles of amplification, after first optimizing these conditions in U87 technical replicates. Gel size selection for 150–700 bp fragments was added to optimize yield. Libraries were equilibrated and sequenced paired-end on Illumina HiSeq 2500, 32 bp read length, at 70–150 mil depth total reads. Raw sequenced reads were aligned to the hg19/GRCh37 reference assembly using Bowtie2 v2.1.0. Peaks were defined using MACS2 (parameters: –f BAMPE –shift -100 –extsize 200). Peaks separated by fewer than 150 bp were merged. Differential peak calling was performed using the DESeq2 R package. GREAT was employed for functional gene set analysis. Using the “basal plus extension association rule setting” in GREAT, each gene was assigned a basal regulatory domain of a minimal distance of 5 kb upstream and 1 kb downstream of the transcription start site (TSS), and an extended regulatory domain up to 1000 kb upstream and downstream of the gene’s basal domain. The HOMER tool suite (v4.8) was used for de novo TF motif discovery, by analyzing differential motif enrichment in GSC tumor-specific and developmentally shared differential regulatory element datasets against all elements (peaks) background. The selection of differentially enriched motifs was further prioritized by selecting those motifs enriched in only one of the two peak set analyses (tumor-specific or developmental) but not in both. For the RNA-seq/ATAC-seq correspondence analysis (Fig. 5a), we first associated each differential peak with its nearest gene using default Homer settings (where each peak is associated with the nearest gene’s TSS). For each gene, the peak with the highest score among all associated peaks was retained and shown in Fig. 5a.

### Transcriptome data preparation and analysis

Whole-transcriptome data were generated via RNA-seq in sham, TEAD1KO, and TEAD4KO cell lines G-13063, G-13306, G-12746, G-16302, and sham and TEAD1KO migration assay spheroids G-13063 (three replicates from separate experiments). cDNA was generated using Clontech SMART-seq v4 (634888) and libraries using Nextera XT (Illumina FC-131–1024). Data were sequenced at 50 bp, 30–40 million paired-end reads/sample, on Illumina HiSeq 2500. Output sequencer files for each sample were subjected to a quality control assessment using the FASTQC package. RNA-seq reads were aligned to the human genome (GRCh38) using STAR with default settings. Gene counts were obtained using the featureCount utility of the subread 5 package. The count data were then rld (rlog transformed counts)-normalized (the rld function transforms the count data to the log2 scale in a way which minimizes differences between samples for rows with small counts, and which normalizes with respect to library size).

Transcriptome analysis was performed on sham/TEAD1KO/TEAD4KO RNA-seq data as well as on previously generated RNA-seq data in acutely sorted E+/E− GBM and NSPC populations (GEO GSE96682, https://www.ncbi.nlm.nih.gov/geo/query/acc.cgi?acc=GSE96682)^17^. Differential expression analysis was performed using the DESeq2 R package, modeling the data with a negative binomial distribution and using Empirical Bayes shrinkage for dispersion and fold change estimation. Functional enrichment analyses were performed using GSEA, DAVID, and Pantherdb tools (see Supplementary references). All RNA-seq tests were FDR adjusted for multiple testing correction.

The expression of selected genes (AQP4 shown) was validated by RT-qPCR. Total RNA was isolated from cell lines via Trizol/chloroform/isopropanol extraction using standard protocol (ThermoFisher, 15596–026), followed by RNA cleanup-and-concentrator kit with DNase treatment (Zymo Research, R1013). cDNA was generated using the High-Capacity RNA-to-cDNA Kit (Life Technologies, 4387406). RT-qPCR reactions were run in duplicates, and Ct values were normalized to the ACTB housekeeping gene.

### TCGA data analysis

GBM RNA-seqV2 normalized count data were downloaded from the Broad Institute Firehose (http://gdac.broadinstitute.org/runs/stddata__2016_01_28/data/GBM/20160128/gdac.broadinstitute.org_GBM.Merge_rnaseqv2__illuminahiseq_rnaseqv2__unc_edu__Level_3__RSEM_genes_normalized__data.Level_3.2016012800.0.0.tar.gz). Primary tumor sample expression data were log2(*x* + 1) transformed, quantile normalized, and corrected for patient age, gender, and batch. Two samples were identified as outliers based on sample clustering and were removed. Genes with missing values or zero variance were removed. This yielded a final dataset of 150 primary GBM samples and 19,942 genes.

Differences in TEAD family expression in GBM samples were tested using one-sided Wilcoxon matched pairs tests. Genes coexpressed with TEAD1 were defined as those with a TEAD1 Spearman’s *ρ* > 0 and BH-adjusted *p* < 0.05. Functional enrichment of correlated genes was carried out using KEGG Pathway gene sets. All analysis was conducted using the R 3.4.1 statistical computing environment.

### CRISPR-Cas9 knockout and overexpression studies

CRISPR-Cas9 gene-editing experiments were carried out using the lentiCRISPRv2 lentiviral system (Addgene, 52961). Guide RNAs (gRNAs) targeting exon 3 of TEAD1, exon 3 of TEAD4, and exon 1 of EGFR were designed using CRISPR Design and cloned into the lentiCRISPRv2 vector (Supplementary Fig. 5). The lentiCRISPR v2 vector with a gRNA insert (5000 ng), the packaging plasmid psPAX2 (3750 ng, Addgene, 12260), and the envelope plasmid pMD2.G (1250 ng, Addgene, 12259) were mixed together and then added to a mixture of 60 μl X-tremeGENE^™^ 9 DNA Transfection Reagent (Roche) and 1 ml OPTI-MEM (Thermo Fisher Scientific, 31985070).

Lenti-X 293T cells (Takara, 632180) were used for transfection and harvesting of the lentivirus at 72 h. Then, cells were infected at 50% confluency with freshly collected CRISPR-Cas9-gRNA lentivirus supplemented with 8 μg ml^−1^ polybrene (Sigma-Aldrich, TR-1003-G). Infected cells were selected in media using puromycin treatment for up to 14 days (2 μg ml^−1^). Uninfected mock control cells were used to ensure uniform cell death within 14 days of puromycin treatment. For overexpression studies, the (Myc-DDK-tagged)-TEAD1, -AQP4, and CDH11 human ORF lentivirus constructs were transfected and purified from Lenti-X 293T cells (Origene RC215492L1, RC204693L1, and RC203810L1, respectively). GBM cells were infected for 12 h and allowed to recover for at least 48 h, prior to analysis.

Successful mutation of infected primary GBM cell lines was confirmed with the Surveyor® Mutation Detection Kit (Integrated DNA Technologies; 706025). Briefly, genomic DNA was extracted from infected cells and the region surrounding the PAM sequence was amplified using the high-fidelity PrimeSTAR GXL DNA Polymerase (Clontech, R050A). Purified PCR products were incubated with the surveyor nuclease and enhancer S with additional MgCl_2_ (0.15 M) for 30 min at 42 °C, following manufacturer’s protocol (Supplementary Fig. 5, Supplementary Table 2 for primer and gRNA sequences). Protein knockout was confirmed by Western immunoblot. For G-13063, each CRISPR-Cas9 gRNA experiment was repeated two independent times, involving separate infections. In each experiment, cells were plated in at least three replicates for sphere and migration assays.

### Cell culture (sphere, proliferation, and migration assays)

Functional validation was performed on patient-derived, low-passaged (less than 20) IDH-wildtype primary GBM cell lines, which were selected for sphere-forming cells by growth on laminin (10 μg ml^−1^) in serum-free NS media supplemented with EGF (20 ng ml^−1^) and bFGF (20 ng ml^−1^) (G-13063 was used in initial experiments; G-13306, G-12746, and G-16302 were used in validation set of experiments). Migratory behavior was assessed using spheroid migration and transwell invasion assays. For spheroid migration, spheres were grown in serum-free NS media in low adherent conditions for 1 week, and then placed in poly-D-lysine (PDL) (10 μg ml^−1^) +/− laminin substrate in serum-free conditions without EGF or bFGF growth factors. Cell migration was assessed at 1 h and 30/36 h by measuring area of confluent migration. To study the migration dynamics, pictures were taken every hour on In Cell Analyzer 2200. For transwell invasion assays, cells were dissociated with Accutase® and counted. About 25,000 live cells were plated in the top chamber of the transwell (Corning, 3470) without growth factors (only containing DMEM/F12, 0.6% D-Glucose, 2 mM L-Glutamine, 1× Antibiotic–Antimycotic, 15 mM HEPES) and allowed to invade through a laminin-coated (20 μg ml^−1^) porous membrane following growth factor and serum attractants placed in the bottom chamber (DMEM/F12, 1× N2, 1× B27, 1× ITS, 0.6% D-Glucose, 2 mM L-Glutamine, 1× Antibiotic–Antimycotic, 15 mM HEPES, EGF (20 ng ml^−1^), bFGF (20 ng ml^−1^), 10% FBS). Percent cell invasion was measured at 24 h by quantitating the number of invasive cells (adherent to the bottom of the membrane) out of total cells plated. For sphere assays, cells were cultured on 96-well low-adherence plates in freshly made NS media with addition of EGF (20 ng ml^−1^) and bFGF (20 ng ml^−1^), at a density of 2.5 c μl^−1^, and their sphere diameter and number were recorded 6 days later. To calculate stem cell frequency, extreme limiting dilution analysis (ELDA) was performed^79^. Briefly, cells were seeded at 1, 10, 50, and 100 cells/well and their sphere-forming capacity was recorded at 21 days (20 wells per condition, ~20 μM sphere diameter cut off for counting). To analyze cell proliferation, cells were seeded in triplicates on laminin-coated wells at 25 K cell density, and were allowed to attach for 12 h. Cells were harvested at 24, 48, and 72 h, detached, and counted using a hemocytometer.

### Orthotopic transplantations

Animal studies were performed in accordance with the ethical standards of the Icahn School of Medicine at Mount Sinai (ISMMS) under an approved Institutional Animal Care and Use Committee (IACUC) protocol. CRISPR-Cas9 sham and TEAD1KO primary GBM cells from G-13063 (2 × 10^5^) were injected stereotactically into the striatum (2 mm right lateral to bregma and 3 mm deep) of 2-month-old male and female mice with B & T cell ICR-Severe Combined Immunodeficiency (SCID) (IcrTac:ICR-Prkdc^SCID^ strain, Taconic). Mice were sacrificed at 3.5 months for histological analysis of tumor infiltration during early xenograft formation. Area of migration was measured using Zen 2 (blue edition).

### Chromatin immunoprecipitation

TEAD1 binding was validated by ChIP using established protocols for TF-ChIP^16,80^. Briefly, chromatin was extracted from freshly snap-frozen primary GBM tissues, gently cross-linked (1% formalin, 10 min), sheared to approximately 150–400 bp (Bioruptor^TM^ Twin, Diagenode), and subjected to immunoprecipitation with antibodies against TEAD1 (BD, 610923, 5 μg per sample), TEAD4 (Santa Cruz, sc-101184, 5 μg per sample), or IgG (12–371, Millipore, 5 μg). The TF/chromatin-associated DNA was purified and quantified by qPCR using primers spanning TEAD1--accessible open chromatin regions in multiple genes, and normalized to input (Supplementary Table 2 for primer sequences).

### Western immunoblot and immunofluorescence

Immunoblotting was performed by standard protocol using the following antibodies: TEAD1 (ThermoFisher, PA5-37075, 1:100, and BD, 610923, 1:100), TEAD4 (Santa Cruz sc-101184, 1:500), EGFR (Millipore, 06–847, 1:1000), pEGFR (Abcam, ab40815, 1:2000), AQP4 (Novus, NBP187679m 1:500), ERK (Santa Cruz, sc-135900, 1:500), pERK (Santa Cruz, sc-81492, 1:500), AKT (Cell Signaling, 4691T, 1:500), pAKT (Cell Signaling, 4060T, 1:500), CDH11 (ThermoFisher, 71–7600, 1:250) and ACTB (ThermoFisher, MA5-15739, 1:10,000), which were diluted in 2.5% BSA in TBS-T solution. HRP-linked anti-mouse (GE Healthcare, NA9311ML) and anti-rabbit (GE Healthcare, NA9341ML) secondary antibodies were used (1:500) to detect immunoreactivity. Main experiments were performed at least two independent times. Representative uncropped blot scans are supplied in Supplementary Fig. 8.

Immunofluorescence analysis was performed on mouse PDX tissue sections (mouse brains were perfused with 4% paraformaldehyde (PFA) and post-fixed in 4% PFA for 24 h prior to analysis). All sections underwent: blocking in 10% normal donkey serum (NDS)/0.5% triton-X (TX) for 1 h at RT; primary antibody incubation (1% NDS/0.25% TX overnight at 4 °C) with TEAD1 (ThermoFisher, PA5-37075, 1:100, and BD, 610923, 1:100), TEAD2 (Abcam, ab196669, 1:100), TEAD4 (Santa Cruz, 101184, 1:100), EGFR (Invitrogen, 280005, 1:100, and Millipore, 06–847, 1:100), Ki67 (Abcam, ab15580, 1:250), and HNA (Millipore, MAB1281, 1:400); and species-appropriate fluorochrome-conjugated secondary antibody incubation in 1% NDS/0.25% TX for 4 h at RT. Nuclear counterstain was done with DAPI (ThermoFisher, D1306, 1:1000). Images were captured on a confocal Zeiss LSM710 microscope.

### Statistics

For all biochemical experiments, mean ± SEM were used in two-tailed unpaired Student’s *t*-test to calculate significance (*p < 0.05, **p < 0.01, ***p < 0.001), unless otherwise indicated. For differential ATAC-seq/RNA-seq data analyses, the Wald test was used to calculate *p*-values, which were FDR-adjusted to <0.05. For TCGA RNA-seq data analyses, one-sided Wilcoxon matched pairs test was used to calculate *p*-values for TEAD1-4 expression and one-tailed Fisher’s exact test was used to calculate *p*-values (BH-adjusted) for TEAD1-correlated enrichment.

## Electronic supplementary material

Supplementary Information

Peer Review Files

Description of Additional Supplementary Files

Supplementary Data 1

Supplementary Data 2

Supplementary Data 3

Supplementary Movie 1

Supplementary Movie 2

Supplementary Movie 3

Supplementary Movie 4

Supplementary Movie 5

## Data Availability

All data generated and analyzed in this study are included in this article (and its Supplementary Information files). TATAC-seq and RNA-seq data generated in this study are deposited in the GEO database under GSE117685. Accession codes or web links of publically available datasets used are provided in the appropriate methods subsections.
