# Supplementary material for: Analysis of chromatin accessibility uncovers TEAD1 as a regulator of migration in human glioblastoma
Source: Nat Commun. 2018 Oct 1;9:4020. doi: 10.1038/s41467-018-06258-2 (PMC6167382; doi:10.1038/s41467-018-06258-2)
Supplement: Supplementary file 1 — Supplementary Information [file 41467_2018_6258_MOESM1_ESM.pdf]

SUPPLEMENTARY INFORMATION

Analysis of chromatin accessibility uncovers TEAD1 as a regulator of migration in human glioblastoma

Tome-Garcia et. al

This file includes:

- **Supplementary Figures 1-8**
- **Supplementary Table 1**
- **Supplementary Table 2**
- **Supplementary References**

Supplementary Figure 1 (relates to Figure 1)

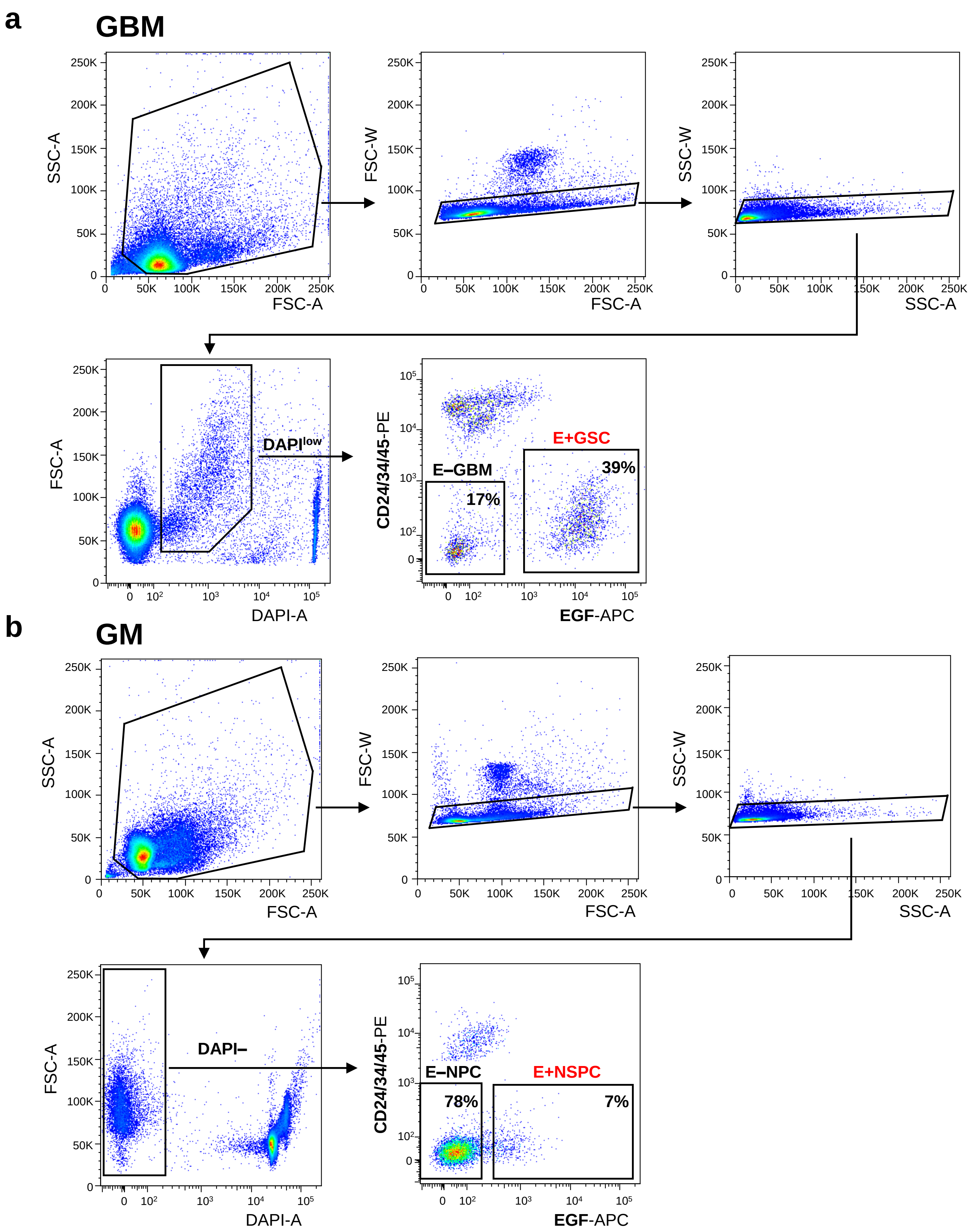

Supplementary Figure 1. Sequential FACS gating strategy to isolate E+GSCs and NSPCs

a-b. Two representative sequential FACS gating strategies are shown, used to isolate EGF-bound (E+) stem cell populations from human GBM (a) and GM (b) tissues, respectively [1,2]. Note that in the case of GBM, cells were isolated specifically from the DAPI^{low} live cell fraction, which we have previously found to be enriched in tumoral cells. The final gating panels for GBM and GM (EGF-APC and CD24/34/45-PE) correspond to panels shown in Figure 1a. SSC=Side scatter; FSC=Forward scatter; A=area; W=width.

Supplementary Figure 2 (relates to Figure 1 and Figure 2)

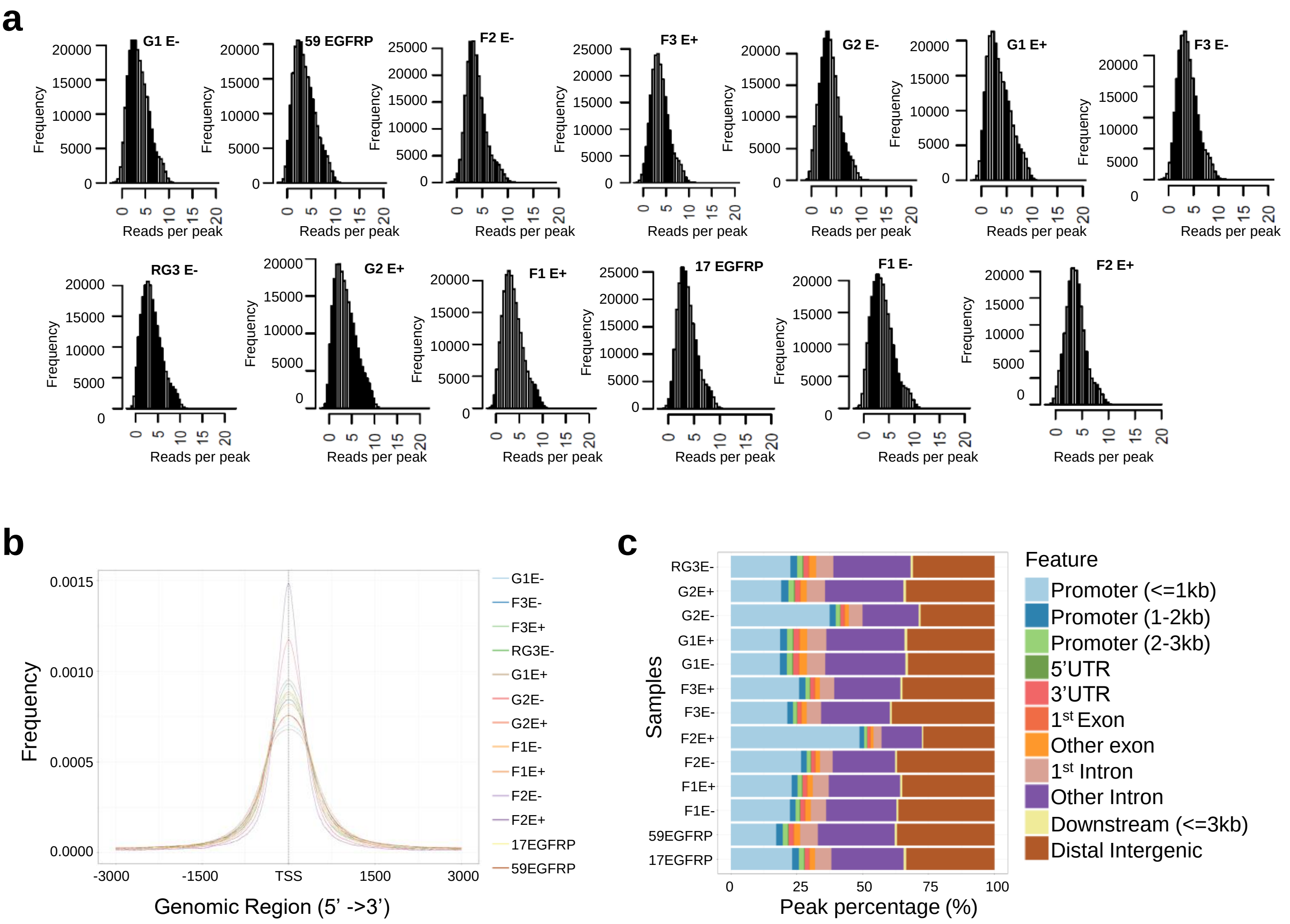

Supplementary Figure 2. Quality control analysis of ATAC-seq data for all samples

a. The frequency distribution of ATAC-seq reads per peak is comparable for all samples.

b-c. The distribution of peaks in all samples centers around the TSS (b) and shows expected enrichment at promoters and other regulatory regions (c). Sample legend: E+NSPCs = F1E+, F2E+, F3E+; E–NPCs = F1E–, F2E–, F3E–; E+GSCs = G1E+, G2E+, 59EGFRP, 17EGFRP; E–GBM = G1E–, G2E–, RG3E–.

Supplementary Figure 3 (relates to Figure 1 and Figure 2)

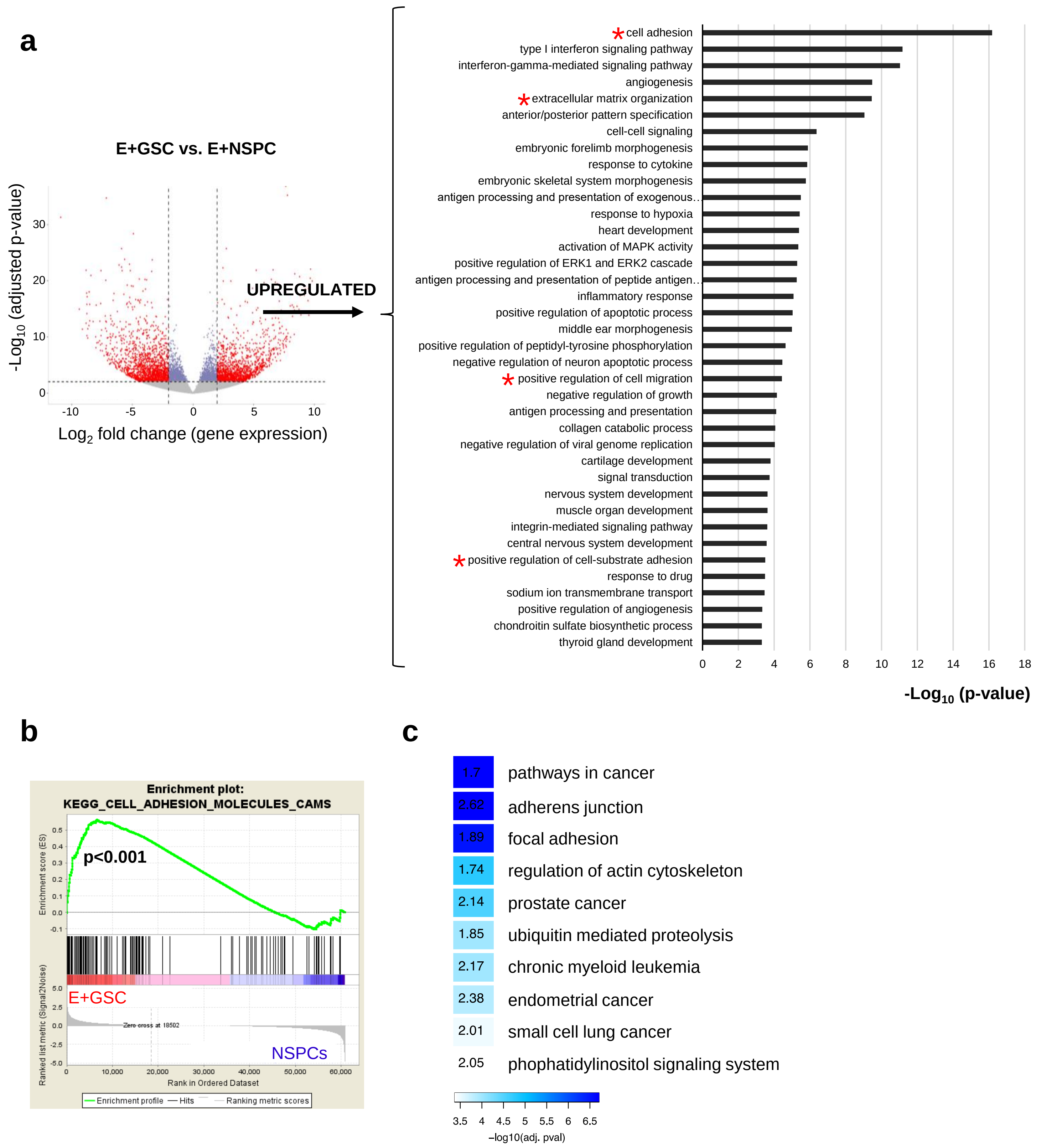

Supplementary Figure 3. Functional enrichment analyses of E+GSC/NSPC RNA-seq data

a. Functional enrichment analysis of differentially upregulated genes (log2 (fold change) = 2; p-adj. < 0.05) in E+GSC vs. E+NSPC RNA-seq dataset (GSE96682) [1] reveals highest enrichment for the "cell adhesion" gene ontology term; other migration-related terms are also significantly enriched (red *) (DAVID tool [3]).
b. The "Adhesion molecules pathway CAMs" KEGG pathway is significantly enriched in E+GSCs compared to NSPC rld-normalized gene expression data (GSEA tool [4]) (all rld-normalized values included).
c. KEGG pathway [5] enrichment of genes co-expressed with TEAD1 curated from the TCGA GBM RNA-seqV2 dataset (n = 150). Top 10 significant terms (BH-adjusted p < 0.05) are shown. Values in cells represent fold enrichment.

Supplementary Figure 4 (relates to Figure 2 and Figure 6)

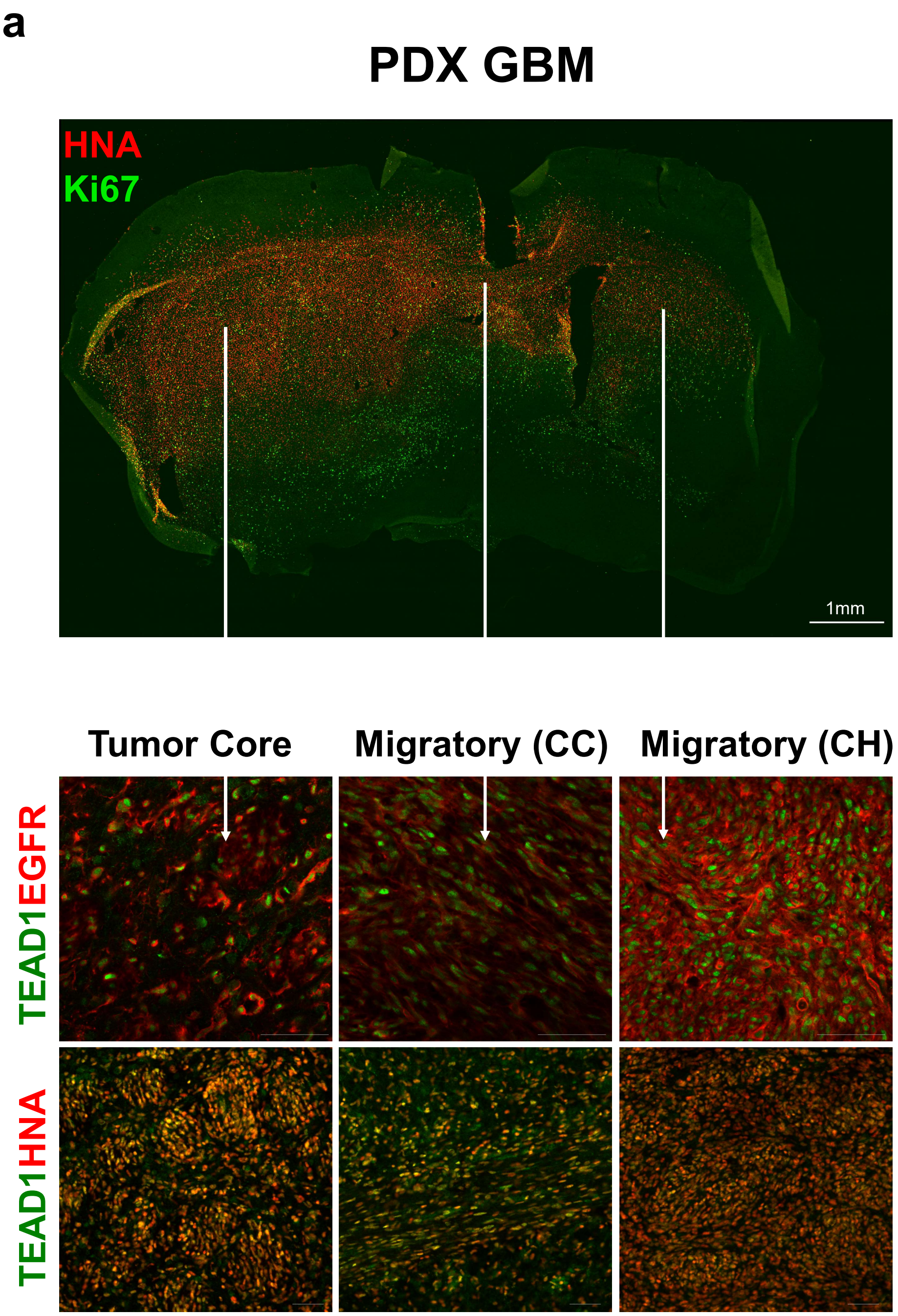

Supplementary Figure 4. TEADs expression in GBM tissues and PDX glioma models

a. Representative immunofluorescence images from previously described patient-derived xenograft (PDX) high-grade glioma [1], showing diffuse TEAD1 expression in grafted human tumor cells (human nuclear antigen, HNA-positive) and frequent TEAD1/EGFR co-expression (CC = corpus callosum, CH = contralateral hemisphere to injection / tumor core).

b. Representative immunofluorescence images from the same PDX sample as above, but immunostained with TEAD2 or TEAD4. TEAD2 and TEAD4 expression is not detected in HNA-positive tumor cells. TEAD2-positive HNA-negative cells are seen at the edge of some PDX gliomas, consistent with infiltrative mouse parenchyma cells.

c. Validation of TEAD4 antibody (Sham vs. TEAD4KO GBM cell line G-13063).

Scale bar = 50μM, unless otherwise specified.

Supplementary Figure 5 (relates to Figure 3)

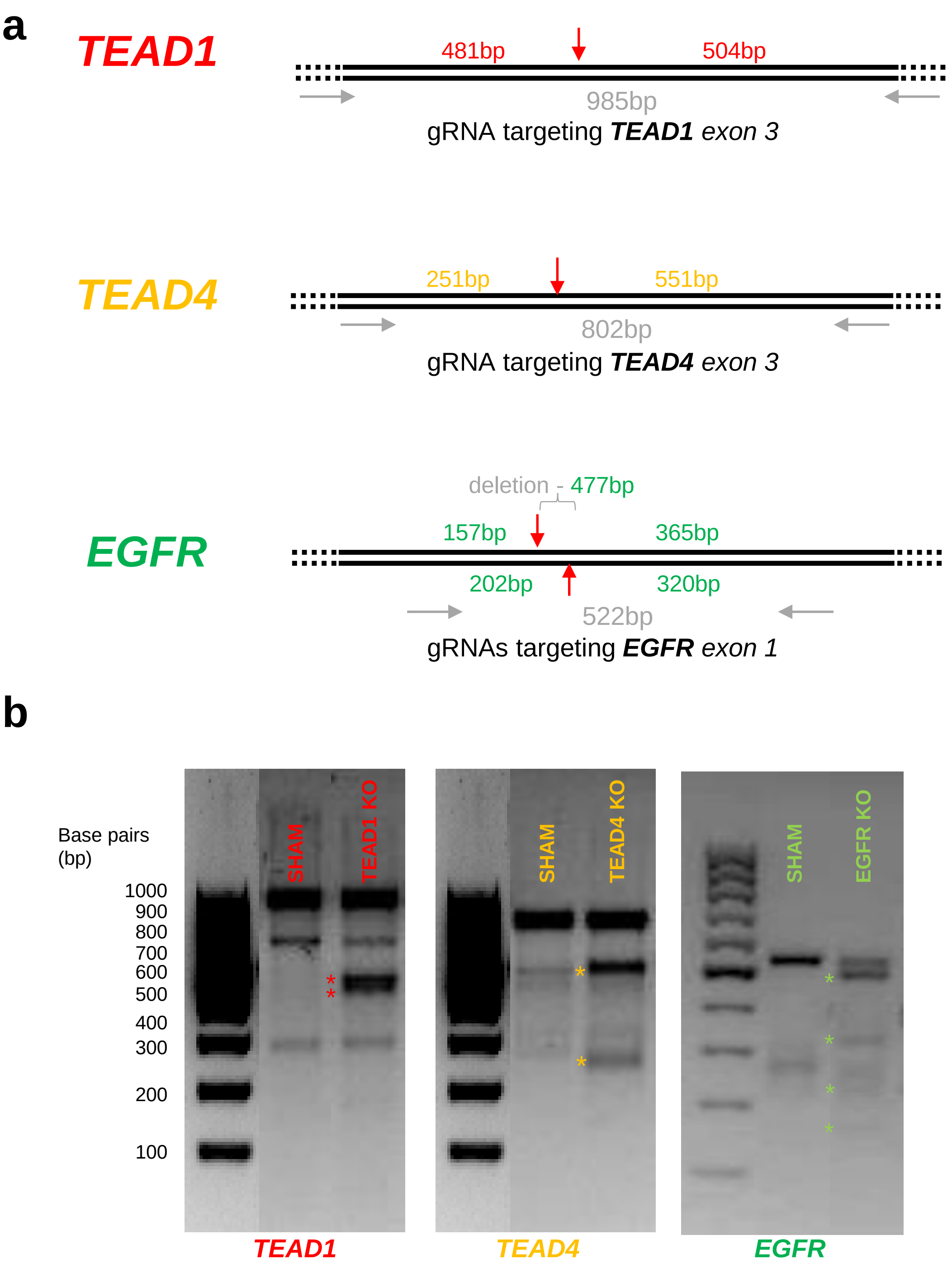

Supplementary Figure 5 (relates to Figure 3) (continuation)

c

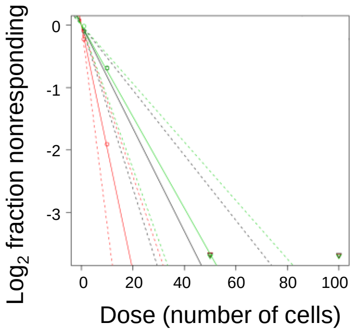

Group	% SC Freq.	P value
SHAM	19.7%	
EGFR KO	8.2%	< 0.05
TEAD1 KO	7.3%	<0.05

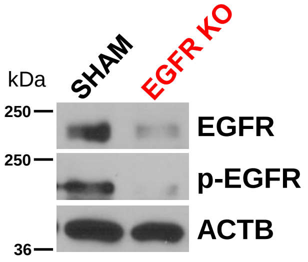

d

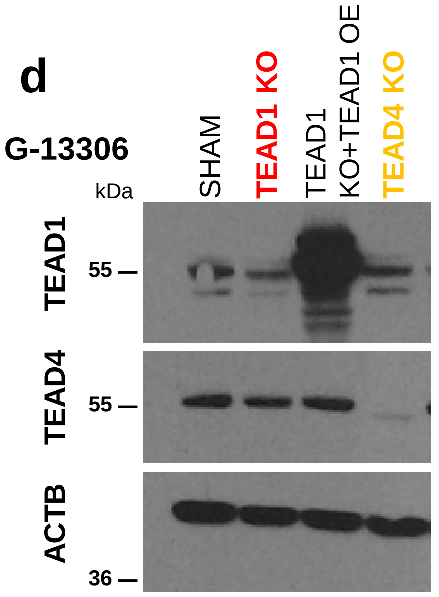

e

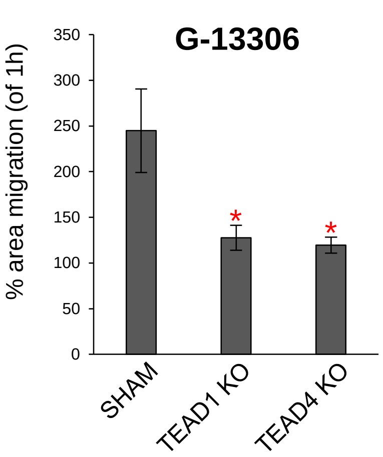

f

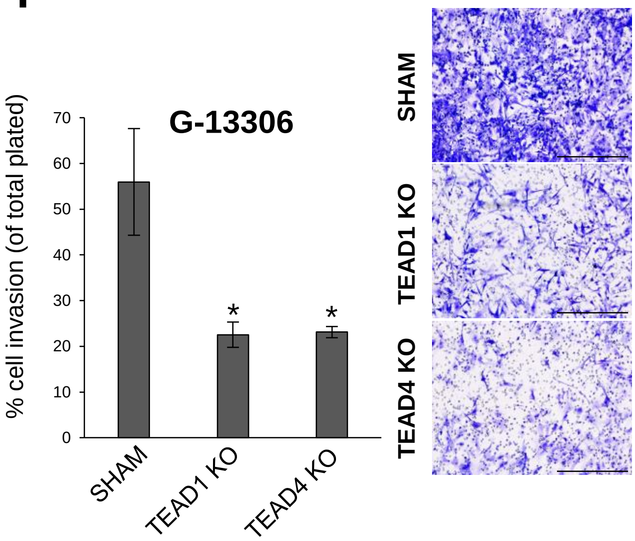

d

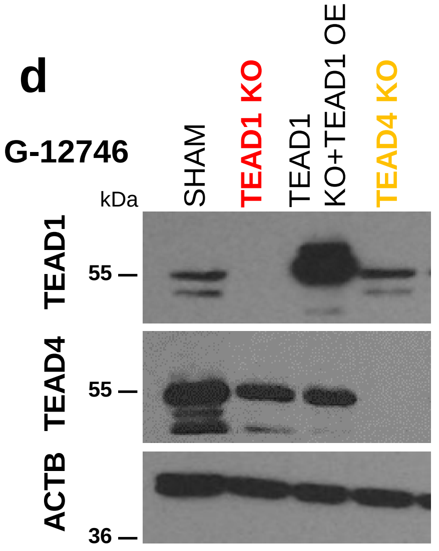

e

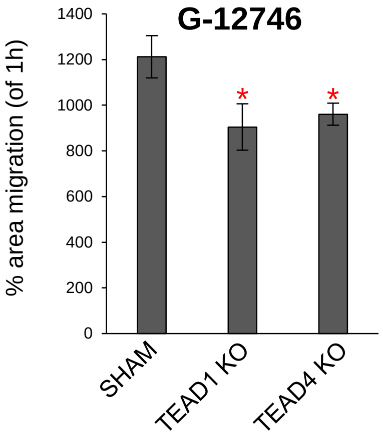

f

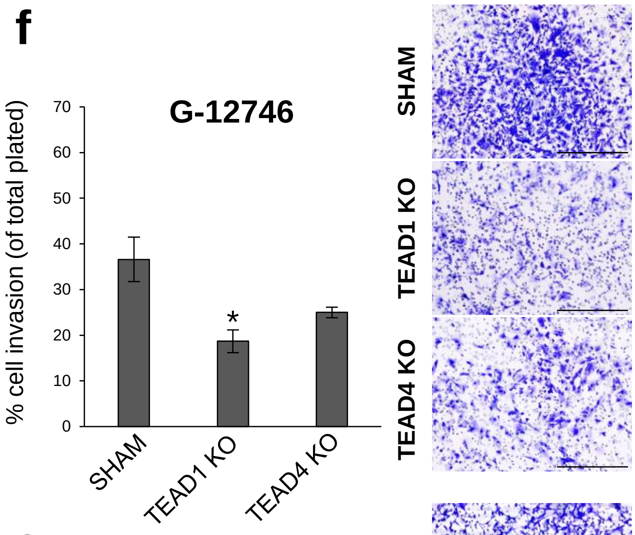

d

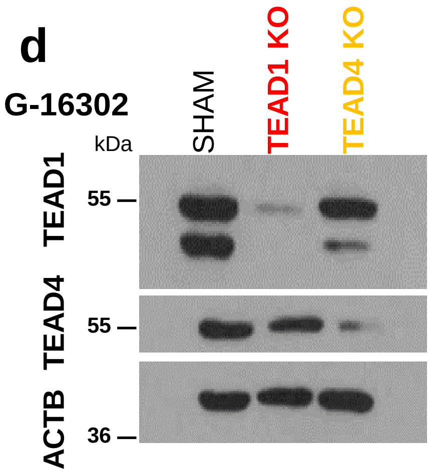

e

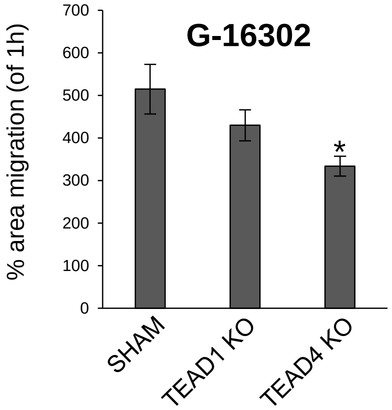

f

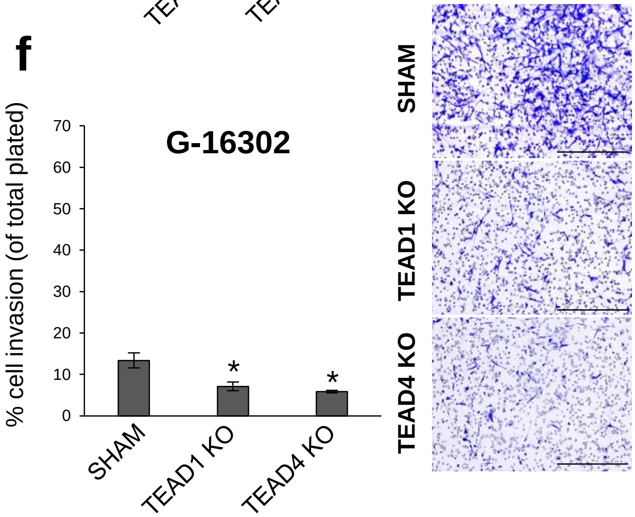

Supplementary Figure 5. Functional characterization of CRISPR-Cas9-mediated TEAD1 or TEAD4 knockouts in primary GBM cell lines

a. CRISPR-Cas9 gene ablation strategy used to knockout TEAD1, TEAD4, and EGFR. Guide (g) RNAs (vertical arrows) target TEAD1 exon 3, TEAD4 exon 3, and EGFR exon 1 (2 gRNAs used). Expected band sizes for cleaved heteroduplexes in Surveyor assay are also shown.
b. Surveyor assay confirms CRISPR-Cas9-generated mutation at TEAD1, TEAD4, and EGFR, in region surrounding PAM sequence. A mismatch-specific DNA endonuclease from the IDT Surveyor® Mutation Detection Kit was used to detect insertion/deletions in heteroduplexes. Cleaved heteroduplexes and deletions are denoted by asterisk (*TEAD1 cleaved heteroduplexes, *TEAD4 cleaved heteroduplexes, *EGFR 45bp deletion and cleaved heteroduplexes).
c. ELDA analysis reveals significantly decreased stem cell frequency at 21 days in TEAD1 knockout G-13063 GBM cells, and as a positive control - in EGFR knockout G-13063 GBM cells, compared to Sham. Pairwise Chi-Square test was used to calculate differences in stem cell frequencies (http://bioinf.wehi.edu.au/software/elda/). Western immunoblot of CRISPR-Cas9-mediated EGFR population knockout is also shown.
d. Western immunoblot confirms population knockout of TEAD1 and TEAD4 after CRISPR-Cas9-mediated gene ablation in three additional patient-derived GBM cell lines (G-13306, G-12746, and G-16302, further described in Supplementary Table 1).
e. Spheroid migration assays show significant decrease in confluent cell dispersion in additional TEAD1-knockout and TEAD4-knockout GBM cell lines, compared to Sham (n=3 wells with multiple NS per well; bars represent mean±SEM, *$p < 0.05$). Red asterisk represents one tailed t-test analysis.
f. Transwell invasion assays show significant decrease in percent cell invasion in additional TEAD1-knockout and TEAD4-knockout GBM cell lines, compared to Sham (n=3 wells; bars represent mean±SEM; *$p < 0.05$). Representative images of transwell invasion chamber membranes are show on the right. Scale bars = 75μM.

Supplementary Figure 6 (relates to Figure 2, Figure 5 and Figure 6)

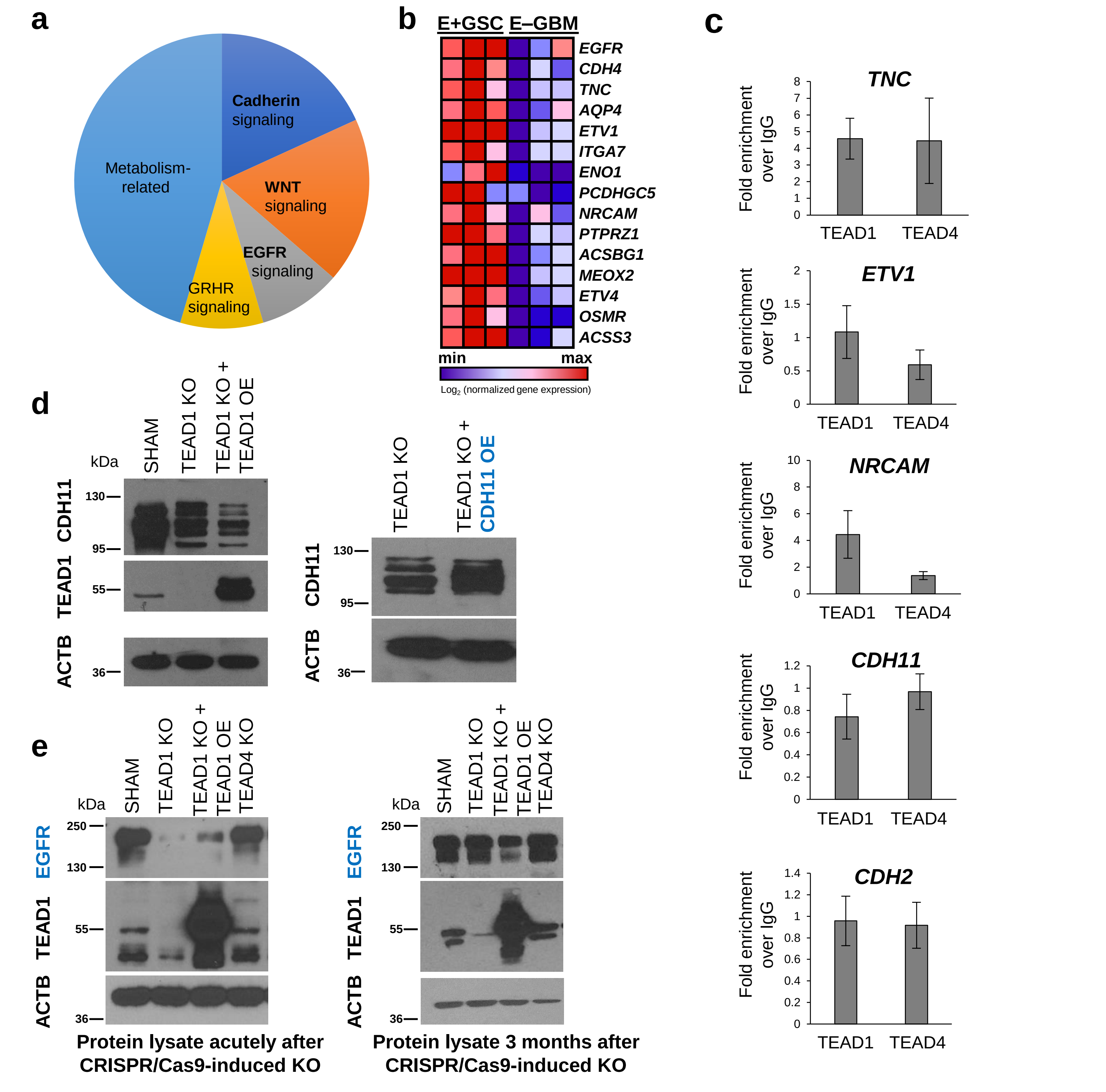

Supplementary Figure 6. Analyses for putative TEAD1 regulatory targets

a. Pie chart depicts top represented pathways (% of gene hit against total number of pathway hits) in the most highly differentially expressed E+GSC genes containing TEAD motifs within tumor-specific open chromatin regions at their proximal promoters [-5kb,+3kb] (PANTHER analysis [6]). Included are all differentially overexpressed genes in E+GSC vs. E–GBM + NSPC RNA-seq analysis (GSE96682) [1] with log2 (fold change) ≥ 3, p-adj. <0.05.
b. Heatmap depicts rld-normalized gene expression values from the above RNA-seq dataset of top-ranked genes with TEAD-associated accessibility peaks at their proximal promoters [–5kb,+3kb] (row-normalized).
c. Chromatin immunoprecipitation (ChIP-PCR) in GBM tissues (n=6) shows no significant enrichment of either TEAD1 or TEAD4 over IgG, at the *TNC*, *ETV1*, *NRCAM*, *CDH11* or *CDH2* genes, assessed at regions with TEAD-associated chromatin accessibility peak(s). Enrichment is expressed as fold increase over IgG, after normalization with 10% input. Bars represent mean±SEM. See supplementary Table 2 for primer sets used.
d. Immunoblot reveals downregulation of CDH11 in TEAD1KO cells, compared to Sham, which is not altered after TEAD1 overexpression (OE) (G-13063 cells) (left). Immunoblot also confirms overexpression of CDH11 after lentivirus infection of G-13063 cells (right).
e. Immunoblot reveals the robust but transient downregulation of EGFR protein in TEAD1KO G-13063 GBM cell cultures.

Supplementary Figure 7 (Relates to Figure 7)

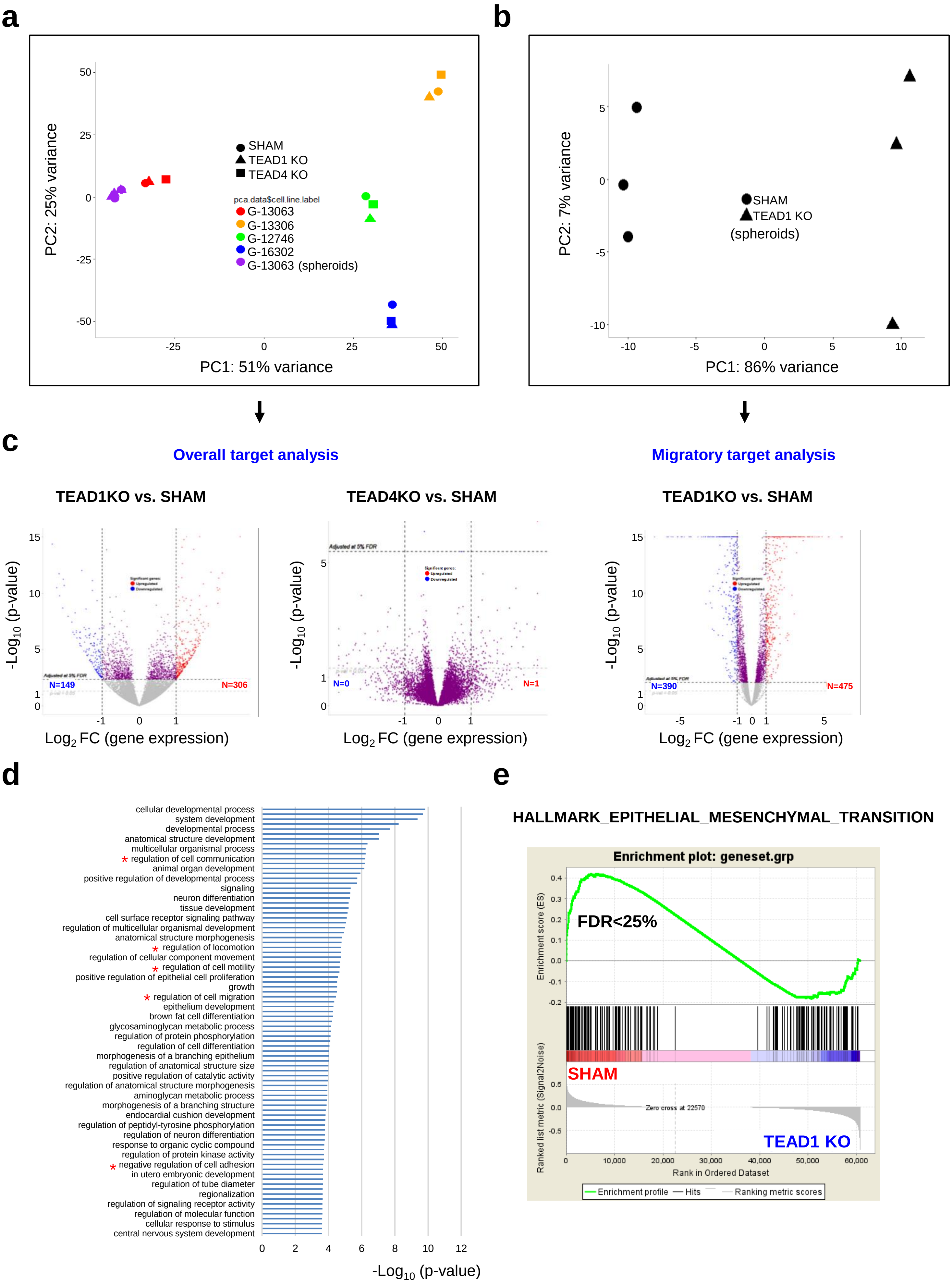

Supplementary Figure 7. Analysis of TEAD1/4-knockout transcriptome signatures

a. Principal component analysis of RNA-seq data from all samples in four cell lines reveals expected separation driven by patient-derived cell line. All TEAD1KO and Sham samples shown here were used in the “overall target” analysis.
b. Principal component analysis of RNA-seq data from migratory Sham vs. non-migratory TEAD1KO spheroids from one cell line, G-13063, shows strong separation by migratory phenotype. All of these samples were used in the “migratory target” analysis.
c. Differential gene expression analyses of TEAD1KO vs. Sham RNA-seq datasets define a TEAD1-associated transcriptome signature. In contrast, differential gene expression analysis of TEAD4KO vs. Sham RNA-seq data does not reveal a significant number of differentially expressed genes to define a TEAD4-associated transcriptome signature.
d. Functional enrichment analysis of significantly downregulated genes in TEAD1KO vs. Sham “overall target” analysis (log2 (fold change) < -1, p-adj. < 0.05) reveals significantly enriched GO terms related to migration, motility, and adhesion, highlighted in red * (Pantherdb tool).
e. Gene Set Enrichment Analysis (GSEA) shows significant enrichment of the “Epithelial-Mesenchymal-Transition” gene set in Sham (vs. TEAD1KO) rld-normalized RNA-seq data (all Sham and TEAD1KO rld-normalized data included).

Supplementary Figure 8 (relates to Figure 3, Figure 5 and Figure 6)

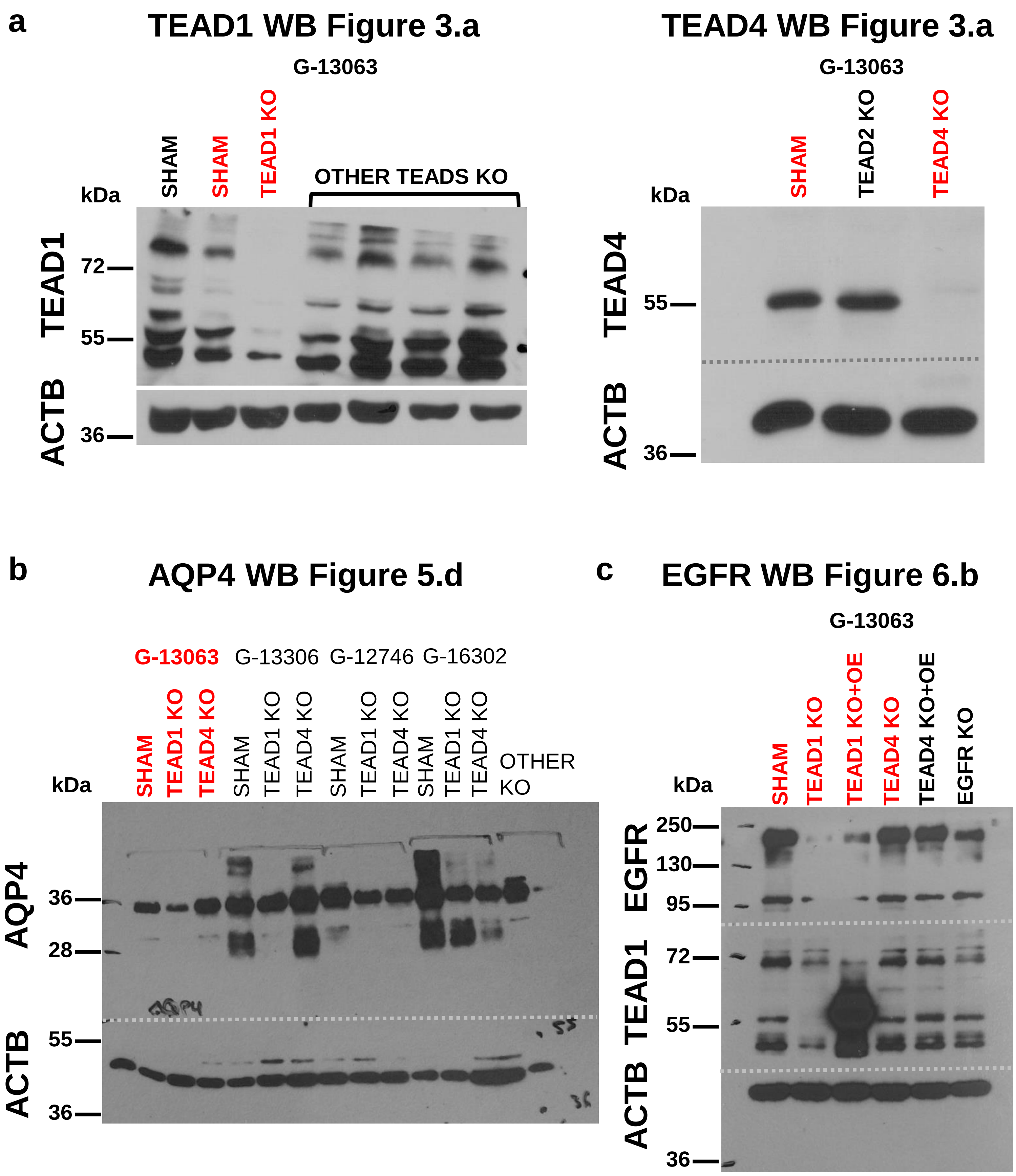

Supplementary Figure 8. Uncropped versions of the most significant Western blots

a-c. Provided are the entire, uncropped immunoblots corresponding to cropped blots shown in Figure 3a (a) , Figure 5d (b) and Figure 6b (c), representative of the most significant experimental findings. Lanes depicted in the main figures are highlighted in red.

Supplementary Table 1 (relates to all figures)

ASSAY/SAMPLE ID	SAMPLE SOURCE	G-13063	G-13306	G-12746	G-16302	G-13181	G-9259	G-9217	G-11074	G-12396	G-11821	G-10006	G-11846	G-11314	G-12048	F-12266	F-10929	F-12437	F-9449
**SAMPLE TYPE (GBM/GM)**	Tissue	GBM	GBM	GBM	GBM	GBM	GBM	GBM	GBM	GBM	GBM	GBM	GBM	GBM	GBM	GM	GM	GM	GM
IDH status (GBM)		wt	wt	wt	wt	wt	wt	wt	wt	wt	wt	wt	wt	wt	wt				
PDGFRA status (GBM)		amp (PN)	wt	wt	wt	wt	wt	wt	wt	wt	wt	wt	wt	wt	wt				
MYC status (GBM)		wt	amp (PN)	wt	wt	wt	wt	wt	wt	wt	wt	wt	wt	wt	wt				
EGFR status (GBM)		wt	wt	inc.CN (CL)	wt	wt	amp,V3 (CL)	amp,V3 (CL)	amp,V3 (CL)	amp (CL)	amp (CL)	amp (CL)	amp,V3 (CL)	amp (CL)	amp,V3 (CL)				
NF1 status (GBM)		mut (MES)	wt	wt	mut (MES)	mut (MES)	wt	wt	wt	wt	wt	wt	wt	wt	wt				
**FETAL DEVELOPMENTAL STAGE**	Tissue															22gw	20gw	19gw	21gw
**ATAC-seq (E+/E– FACS)**	Tissue						X	X	X	X	X					X	X	X	
**ATAC-seq sample ID**	Tissue						59 EGFRP	17 EGFRP	G1E– G1E+	G2E– G2E+	RG3E–					F1 E– F1 E+	F2 E– F2 E+	F3 E– F3 E+	
**RNA-seq (E+/E– FACS)**	Tissue											X	X	X	X	X		X	X
**RNA-seq sample ID**	Tissue											GBM2 E–E+	GBM1 E–E+	GBM1 E–	GBM3 E–E+	GM3 E–E+		GM2 E–E+	GM1 E–E+
**ChIP-PCR**	Tissue	X	X			X	X	X	X	X									
**Transwell invasion**	Cell line	X	X	X	X														
**Sphere migration**	Cell line	X	X	X	X														
**Sphere growth**	Cell line	X																	
**Sphere size**	Cell line	X																	
**ELDA**	Cell line	X																	
**Cell proliferation**	Cell line	X																	
**TEAD1, AQP4, CDH11 OE rescue**	Cell line	X																	
**EGFR immunoblots**	Cell line	X																	
**pERK/pAKT Immunoblots quantification**	Cell line	X	X	X	X														
**In vivo xenotransplantation**	Cell line	X																	
**RNA-seq "overall target"**	Cell line	X	X	X	X														
**RNA-seq "migratory target"**	Spheroids	X																	

wt = wild type
mut = mutation
amp = amplification present
V3 = Presence of EGFR V3 mutation
inc.CN = increased copy number
CL = Classical GBM type
PN = Proneural GBM type
MES = Mesenchymal GBM type
gw = gestational weeks

Supplementary Table 1. Sample information

Supplementary Table 2 (relates to Figure 3, Figure 5, Figure 7)

Surveyor Primers	Forward/ Reverse	Sequence (bp)
TEAD1	Forward	5’-AGATTATCCAGCTTCTGCCCA-3’
	Reverse	5’-GCAATCAAGGGATTCGGGAG-3’
TEAD4	Forward	5’-AGTTCTTCCACTGCTCACCG-3’
	Reverse	5’-CTGCCTTGCAGAGCAGGTAT-3’
EGFR	Forward	5’-CAGACCGGACGACAGGC-3’
	Reverse	5’-AGAAAGTTGGGAGCGGTTCG-3’
**gRNAs for CRISPR**	Forward/ Reverse	Sequence (bp)
TEAD1 (gRNA targets exon 3)	Forward	5’-CACCGGAAAGCTTTGCTCGATGTCG-3’
	Reverse	5’-AAACCGACATCGAGCAAAGCTTTCC-3’
TEAD4 (gRNA targets exon 3)	Forward	5’-CACCGCTCGCCATCTACCCGCCCTG-3’
	Reverse	5’-AAACCAGGGCGGGTAGATGGCGAGC-3’
EGFR (gRNA targets exon 1)	Forward	5’-CACCGTCCTCCAGAGCCCGACTCGC-3’
	Reverse	5’-AAACGCGAGTCGGGCTCTGGAGGAC-3’
EGFR (gRNA targets exon 1)	Forward	5’-CACCGGCGACCCTCCGGGACGGCCG-3’
	Reverse	5’-AAACCGGCCGTCCCGGAGGGTCGCC-3’
GFP SHAM	Forward	5’-CACCGGGGCGAGGAGCTGTTCACCG-3’
	Reverse	5’-AAACCGGTGAACAGCTCCTCGCCCC-3’
**RT-qPCR primers**	Forward/ Reverse	Sequence (bp)
AQP4	Forward	5’-ACTGGTGCCAGCATGAATCC-3’
	Reverse	5’-GGGCCCAACCCAATATATCCAA-3’
ACTB	Forward	5’-TCAAGATCATTGCTCCTCCTGAG-3’
	Reverse	5’-ACATCTGCTGGAAGGTGGACA-3’

Supplementary Table 2 (relates to Figure 3, Figure 5, Figure 7) (continuation)

**ChIP-PCR primers**	Forward/ Reverse	Sequence (bp)
EGFR DISTAL PROMOTER	Forward	5’-GAGCGCTACCCAGGAATGTT-3’
	Reverse	5’-TCCTCTGTCTCCTCCTCAACC-3’
EGFR PROXIMAL PROMOTER	Forward	5’-TGGGACACTTAGCCTCTCTAA-3’
	Reverse	5’-TCCGAGGTGGTGCTCTAA-3’
EGFR INTRON 2 (IN2)	Forward	5’-TTGCAGATCGTGGACATGCT-3’
	Reverse	5’-GACGCCCAGTTGAACCCTAA-3’
CDH4	Forward	5’-CCTCCCCATCCTAAAACCAC-3’
	Reverse	5’-GCCCTGGTATGGAGCATCTA-3’
AQP4	Forward	5’-GTGAGGAAATGCAGTGCCAA-3’
	Reverse	5’-CCCCATAGCAAACTAAGGGCT-3’
TNC	Forward	5’-GGAAGGAACCCATTTGCAT-3’
	Reverse	5’-AGGCGGGAATTCCTACTTTC-3’
ETV1	Forward	5’-ACTATGGACAGCAAAGACAGACC-3’
	Reverse	5’-AGTTCCCGCTCAAAATGCTT-3’
NRCAM	Forward	5’-GCATTGTCATTCAGCTTCCAGG-3’
	Reverse	5’-AGGCAAGGCACAGATTTCAAC-3’
CDH11 peak72095	Forward	5’-TTGAAAGGATCGAAGCATGTCAG-3’
	Reverse	5’-GTTCAGAGGAGACAGAGGCTT-3’
CDH11 peak72146	Forward	5’-AGCACAGCCATAGCCATAGA-3’
	Reverse	5’-CAGGGAAGGCACACAGTAGAA-3’
CDH11 Promoter_1	Forward	5’-TTGCTTTGCGTTAGTGAAGCC-3’
(Shown in Supplementary Fig.6c)	Reverse	5’-GGTTGGGGCTTCTTGGAATG-3’
CDH11 Promoter_2	Forward	5’-CGCCAAGACATTCTCTTCCTG-3’
	Reverse	5’-CCCTTCCTCTCGCCTGGTT-3’
CDH11 Intron1.1	Forward	5’-CTCGTCCCTGCTGGGTAGTA-3’
	Reverse	5’-TGCACCATGACCTTCGTGAT-3’
CDH11 Intron1.2	Forward	5’-AATAAAACGGCACTGCCAGC-3’
	Reverse	5’-TCCATCTGCCAGCCTGAATG-3’
CDH2	Forward	5’-GGAATGAGAAGAGACGGTTCC-3’
	Reverse	5’-TGAAGTTGTGGGTGTGTGCC-3’

Supplementary Table 2. Primer and gRNA sequences used for Surveyor assay, CRISPR-Cas9 knockout, ChIP-PCR, and RT-qPCR

Supplementary References

1. Tome-Garcia, J. *et al.* Prospective Isolation and Comparison of Human Germinal Matrix and Glioblastoma EGFR+ Populations with Stem Cell Properties. *Stem cell reports*, doi:10.1016/j.stemcr.2017.03.019 (2017).
2. Tome-Garcia, J. D., F.; Tsankova, N. M. FACS-based Isolation of Neural and Glioma Stem Cell Populations from Fresh Human Tissues Utilizing EGF Ligand. *Bio-protocol* **7**, e2659 (2017).
3. Huang da, W., Sherman, B. T. & Lempicki, R. A. Systematic and integrative analysis of large gene lists using DAVID bioinformatics resources. *Nature protocols* **4**, 44-57, doi:10.1038/nprot.2008.211 (2009).
4. Subramanian, A. *et al.* Gene set enrichment analysis: a knowledge-based approach for interpreting genome-wide expression profiles. *Proceedings of the National Academy of Sciences of the United States of America* **102**, 15545-15550, doi:10.1073/pnas.0506580102 (2005).
5. Kanehisa, M., Furumichi, M., Tanabe, M., Sato, Y. & Morishima, K. KEGG: new perspectives on genomes, pathways, diseases and drugs. *Nucleic acids research* **45**, D353-D361, doi:10.1093/nar/gkw1092 (2017).
6. Thomas, P. D. *et al.* PANTHER: a library of protein families and subfamilies indexed by function. *Genome research* **13**, 2129-2141, doi:10.1101/gr.772403 (2003).
