# Supplementary material for: Analysis of chromatin accessibility uncovers TEAD1 as a regulator of migration in human glioblastoma
Source: Nat Commun. 2018 Oct 1;9:4020. doi: 10.1038/s41467-018-06258-2 (PMC6167382; doi:10.1038/s41467-018-06258-2)
Supplement: Supplementary file 3 — Description of Additional Supplementary Files [file 41467_2018_6258_MOESM3_ESM.pdf]

Description of Additional Supplementary Files

File Name: Supplementary Movie 1
Description: Depicts the normal dynamic dispersion of (CRISPR-sham) spheroid GBM cells from 0 to 30hrs. The movie is constructed from 30 images at 1-hour interval taken of live spheroid cells incubated within the In Cell Analyzer 2200.

File Name: Supplementary Movie 2
Description: Depicts severe deficits in cell dispersion of spheroids generated from CRISPR-TEAD1 knockout GBM cells using similar parameters to above. Cells attempt to disperse away from the spheroid core but quickly retract back into the core.

File Name: Supplementary Movie 3
Description: Depict the partial rescue of cell dispersion properties after lentivirus overexpression of TEAD1 (movie#3), AQP4 (movie#4) and CDH11 (movie#5) in TEAD1KO spheroids using similar parameters to above.

File Name: Supplementary Movie 4
Description: Depict the partial rescue of cell dispersion properties after lentivirus overexpression of TEAD1 (movie#3), AQP4 (movie#4) and CDH11 (movie#5) in TEAD1KO spheroids using similar parameters to above.

File Name: Supplementary Movie 5
Description: Depict the partial rescue of cell dispersion properties after lentivirus overexpression of TEAD1 (movie#3), AQP4 (movie#4) and CDH11 (movie#5) in TEAD1KO spheroids using similar parameters to above.

File Name: Supplementary Data 1
Description: **Differential chromatin accessibility peaks and Homer results.**

This data file contains the differential chromatin accessibility peaks inthe Tumor GSC-specific and Developmentally shared analyses. The four tabs contain the following peaks, in order from left to right: 1) All GSC-specific peaks derived from the differential accessibility analysis of E+GSCs vs. E–GBM + NSPCs ATAC-seq data; 2) TEAD-associated GSC-specific peaks derived from the differential accessibility analysis of E+GSCs vs. E–GBM + NSPCs ATAC-seq data; 3) All developmentally shared peaks derived from the differential accessibility analysis of E+GSCs + E+NSPCs vs. E–GBM; 4) Homer de novo TF motif datasets with significantly enriched motifs within open chromatin regions obtained from the two differential accessibility analyses, GSC-specific and Developmentally shared.

File Name: Supplementary Data 2
Description: **Correlation analysis for TEAD1-coexpressed TCGA GBM genes.**

This file contains positive and negative correlation data for GBM gene coexpression with TEAD1, analyzed using the TCGA RNA-seqV2 dataset (n=150). Genes coexpressed with TEAD1 were defined as those with a TEAD1 Spearman's correlation coefficient rho > 0 and BH-adjusted p value < 0.05, calculated using one-tailed Fisher's exact test.

File Name: Supplementary Data 3
Description: **Differentially expressed genes in TEAD1KO vs. Sham RNA-seq datasets.**

This data file contains differentially expressed genes (p-adj. < 0.05) in two different CRISPR/Cas9-induced TEAD1-knockout (TEAD1KO) vs. Sham RNA-seq data analyses (from left to right tabs): 1) TEAD1KO vs. Sham overall target analysis including all TEAD1 and Sham cells and spheroid samples (n=7; 4 biological replicates from G-13063, G-13306, G-12746, G-16302 cells plus 3 replicates from three independent spheroid migration experiments with G-13063 cells); 2) TEAD1KO vs. Sham migratory target analysis (n=3 replicates from three independent spheroid migration experiments with G-13063 cells).
