# Supplementary material for: Analysis of chromatin accessibility uncovers TEAD1 as a regulator of migration in human glioblastoma
Source: Nat Commun. 2018 Oct 1;9:4020. doi: 10.1038/s41467-018-06258-2 (PMC6167382; doi:10.1038/s41467-018-06258-2)
Supplement: Supplementary file 2 — Peer Review Files [file 41467_2018_6258_MOESM2_ESM.pdf]

Reviewer #1 Expert in glioblastoma genomics:

Summary. Tome-Garcia et al extend their earlier findings (Stem cell Reports May 9, 2017) of the enrichment of stem cell populations from germinal matrix and from glioblastoma tissue to an in depth interrogation of epigenetic events distinguishing proliferation and invasion behavior of malignant glioma stem cells. Comparing chromosome accessibility mapping between stem cells (EGF binding cells) isolated from germinal matrix (autopsy) and GBM (freshly resected tumor tissue), distinct regulatory signatures were uncovered. Transcription factor motif discovery elevated TEAD family members as prioritized candidates (distinct from stem cell candidates) as tumor-specific (invasion) instigators, aligning to EGFR expression as a prominent, glioma-associated driver under TEAD regulation. CRISPR knockdown of TEAD family members singled-out TEAD1 as the family member most responsible for EGFR-driven GBM invasion (dispersion and infiltration) by patient-derived GSCs. Functional outcome was confirmed by chromosome immunoprecipitation of TEAD1 binding at an upstream regulatory locus of EGFR. TEAD1 and TEAD4 are important TF mediators of glioma pathology (invasion and proliferation, respectively), while also elevating EGFR as a directly-regulated motogenic and mitogenic driver of glioma pathobiology.

Critique.
Tome-Garcia et al leverage a recent publication from their team where brain stem cells from normal (germinal matrix) and glioblastoma can be studied for shared and distinct epigenetic events that underscore glioma pathology, specifically stem cell self renewal and cell dispersion/invasion. The experimental design, to compare/contrast EGF+ cells from human GM and GBM, absent cell culture propagation, is innovative and (likely) incisive. While the ideal comparison would have been cognate representation of GM and GBM from the same individual, the executed study of allogenic pairs is a best approximation, strengthened by the clustering of chromosome accessibility marks within the different GM specimens distinct from GBM cases. Delivering the unique results of ATAC Seq data on GBM stem cells and GM stem cells the manuscript is attractive.

Fig 1 illustrates the cell sorting outcome for populations that do/do not bind EGF from GM and GBM fresh isolates, and the consequent comparisons of differentially accessible peaks that are proximal to genes (likely the least rigorous assignment of mechanistic association between chromosome accessible measurement and gene expression), but nonetheless uncovering stem cell proliferative related (GO Term enrichment) genes when comparing EGF binding stem cells (GM and GBM) with EGF non-binding GBM cells (posited as "developmental associated" genes). The comparison of genes proximal to chromosome accessible regions in GBM CSCs (EGF binding) distinct from GM EGF binding and GBM non-EGF binding cells uncovered genes whose most statistically significant GO terms are enriched for EGFR signaling and cell invasion behaviors. It would be instructive to report how many genes were associated with 10,509 accessible peaks in E+GSC cells.

Inferring chromosome accessible regions to transcription factor motifs using HOMER de novo motif-discovery algorithm is shown in Fig 2. 26 tumor-specific motifs were uncovered, while 27 motifs shared between GM and GBM EGF binding (stem) cells were noted. Precisely how these listed TFs were prioritized is unclearly described, and the grooming to the top candidate TFs ("…exclusively enriched in only one of the [chromosome accessible] peaks…") is very unclear; likely making this discovery unreproducible by another lab. The comparison in Fig 2C (between E+GSC and E-GBM seems misdirected relative to the text, "TEAD1 was the most highly expressed member of the TEAD family in these GBM cells (Fig 2c)…". The heatmap shows almost no difference in TEAD1 exprssion (fig 2c), and the difference in Fig 2d (~9.7 to 10.5) while being statistically different numerically, is of dubious persuasion. How does this data qualify elevation above the other TF candidates? Since gene expression from the different cell populations already exists (Ref 16), why not show the comparison of the other promising candidates as well (similar to Fig 2d)?

The described agreement between Fig 3A and Fig 2c (expression patterns of TEAD family members in TCGA versus the E+GSC) is unpersuasive in viewing the data shown. Along with the immunofluorescent images in Suppl fig 2 for TEAD1, TEAD2 and TEAD4, the declaration (in Results Section 3) that, "…this analysis prioritized TEAD1 as the most highly expressed TEAD family member in GBM, and implicated its relevance in tumor migration, warranting further functional validation in cGSCs" seems to overstate the presented data. Genes co-expressed with TEAD1 in TCGA GBM samples (Spearman's correlation BH-adjusted $p<0.05$) being highly enriched for terms related to cell migration and cell adhesion (Fig 3B) do not reach a persuasive level warranting inclusing of Fig 3 in the main text of the manuscript.

Fig 4 is a compelling depiction of a role of TEAD1 in GBM migration, invasion, and self renewal in the limited sample set studied.

Fig 5A is inadequately described, leading to confusion as to its message. Fig 5B does not convey a coherent elaboration of the findings; this warrants either better integration to the evolving description of discovery, or elimination. Fig 5C has 14 rows of heatmapped squares, but the assigned genes number only 13; this discrepancy should be readily remedied. The election to focus solely on EGFR (p=1.89e-2) compared to ITGA7 (p=6.47e-06) or other more statistically significant edges (fig 5d) should be better explained.

Fig 6 portrays results linking TEAD1 as a TF controlling expression of EGFR. The closing statement in the Results section, "…TEAD1 as an important TF regulator of the migratory phenotype in primary GSCs, and strongly implicates EGFR as one of its downstream targets" is supported by the data, but is nonetheless overly exclusive of the other associated genes in Fig 5, which are not further developed in this manuscript.

The Discussion is a careful retelling of the results, integrating them into a glioma biology framework where TEAD1 is portrayed as a TF regulating GBM invasion and TEAD4 playing a role in GBM self-renewal (stem like behavior). The other associated genes are called out as meaningful but "beyond the scope of this study". The closing paragraph in the Discussion borders on overstatement, since TEAD1-associated regulation of EGFR does not particularly guide "…us a step closer to targeting the diffusely infiltrative nature of this fatal disease".

The Methods section is reasonably detailed to enable reproduction of the experiments with the exception of the critique of the data in Fig 2 (above).

Reviewer #2 Expert in glioblastoma epigenetics:

Tome-Garcia et al's manuscript titled "Chromatin accessibility uncovers TEAD1 as a regulator of migration and EGFR expression in human glioblastoma" wished to address the specific chromatin status and binding transcription factors (TFs) in glioma stem cells (GSCs). Using the ATAC-seq technology, they identified the regulatory GBM signatures, one of which was tumor-specific related to migration. TF motif analysis prioritized the TEAD family at tumor-specific regulatory regions: the most frequent representation at the epidermal growth factor receptor (EGFR) locus. They concluded that TEAD1 as an important TF regulator in tumor migration, and EGFR as one of the direct targets of TEAD1. This study endeavored to clarify the interaction between chromatin signature and TFs in gliomagenesis and showed some interesting data. However, the paper has significant flaws in the validity of the approach, interpretation of the data and the quality of the presentation.

Specific comments:
1. Detail of their experiments are not well explained. Although authors described that the protocol was optimized using three technical replicates for 25K cells, how consistent between each data set

is? They appeared to examine multiple GBM cells and NSPCs. Are they derived from multiple patients? How many patients were examined? How consistent the ATAC-seq data between each GBM patient? These information are mandatorily required in detail to draw unbiased results.
2. Since the study endeavor to show the importance of TEAD1 during gliomagenesis, genome-wide TEAD1 binding (i.e. ChIP-seq) should be shown in addition to ATAC-seq data in E+/- GBM cells, GSCs, and NSPCs. Instead, they just examined the RNA-seq and ATAC-seq datasets, which were generated from the similar samples in their previous study. They identified EGFR as a TEAD1 target using expression data and motif analysis. However, these sets of analyses may suffer from misinterpretation. In addition, they need to perform RNA-seq expression analysis using the cells with and without TEAD1 (i.e. TEAD1 knockout cells). This study lacks these important experiments to validate their in silico analyses.
3. As authors described, activity of TAZ-TEAD4 has previously been implicated in GBM cell motility and mesenchymal transformation. Although authors claimed that the specific role of TEAD1 in regulating a pro-migration GSC, they failed to show the specific and mechanistic roles of TEAD1-EGFR in gliomagenesis in the study. What is the mechanisms down-stream of TEAD1-EGFR, which affect the GBM cell features, especially stemness and migration? Which molecules are involved? These mechanistic analyses are lacking in the study.
4. Authors need to CRISPR-Cas9-mediated ablation of TEAD1 in E+/E–GBM cells.
5. In vivo functional analysis of TEAD1 is required. For example, conditional CRISPR-Cas9-mediated ablation of TEAD1 in orthotopic xenotransplantation model may help understanding the TEAD1 function in vivo.

In summary, the concepts of the study may be important in the field. However, unfortunately, the study lacks rigorous experiments and may suffer from overinterpretation. The authors' conclusions are based on, in this reviewer's opinion, an unreliable biological system. Consideration of mechanistic data may improve the study

Reviewer #3 Expert in glioma and neuronal stem cells:

In this manuscript, the authors used Chromatin accessibility mapping via ATAC-seq to define two regulatory GBM signatures—a developmentally shared one related to stem cell proliferation, and a tumor-specific one related to migration. They further focused on the migratory signature and transcription factor (TF) motif discovery and the TEAD family at tumor-specific regulatory regions. To further validate the role of TEAD1 in GBM migration and further explore its relationship to EGFR, they generated TEAD1, TEAD4, and EGFR knockouts in patient-derived cultured GBM stem-like cells (CGSCs) using CRISPR-Cas9 technology. The authors conclude that eliminating TEAD1 or TEAD4 diminished migratory properties in CGSCs, mimicking the EGFR knockout effect, and repressed several putative TEAD-targets, including EGFR. They further suggested that chromatin immunoprecipitation data confirmed the specific binding of TEAD1 at an upstream regulatory locus of EGFR.

Overall comments:

This study does not convincingly show that TEAD1 is a key regulator of the invasive nature of GBM or that it is a direct regulator of EGFR expression

Major issues:

This paper highlights the infiltrative nature of GBM as a primary cause for its poor prognosis and concludes that there is a major role of TEAD1 in this process, as well as a role for TEAD1 in regulating EGFR expression.

1. However, this is not convincingly shown in the data presented in the manuscript:

- Fig 4C shows that TEAD1 does not have a significant effect on GSC invasion.
- No differences in TEAD1 expression were seen in tumor core compared to areas of infiltration in Supp. Fig 2. The authors state "we did not observe significantly higher protein expression at the infiltrative tumor element"
- No significant binding of TEAD1 over IgG control in ChIP-PCR to the promoter of EGFR was demonstrated

2. It is often unclear exactly which samples were being used for various experiments. Multiple statements throughout the manuscript describe the use of "similar samples" or "similar cells". It would have been useful to understand which samples were used for experiments in order to allow for critical assessment and interpretation of the data presented in the manuscript.

3. Cell growth was not addressed in any of the experimental data presented throughout the paper. This is surprising considering the well-described role of EGFR on cell proliferation in GBM.

4. The authors describe the use of EGF ligand conjugated to APC for flow sorting for the E+GSC samples. Mutational status of the lines used in experiments (relevant to point 2 above) was not provided. The reviewer also questions how an EGFRvIII sample can be sorted using the EGF-APC condition since EGFRvIII does not bind the EGF ligand.

Minor issues:

1. Intro Paragraph 2
"Epigenetic plasticity is critical in maintaining the stem-cell state during normal development…"
- This statement should be clarifies and a reference specifically describes this phenomenon provided.
- The description of "tumor-specific" analysis is not clear as the analysis compares GSCs to GBM and NSPCs, when comparing to GBM it is therefore not tumor-specific.

2. Results Paragraph 1.

"GBM tumors differ substantially from neural progenitors in their malignant spread and progression."
- Statement is not clear.

Missing reference in Results section 1.
- After "Consistent with previous reports, the developmentally shared GSC/NSPC peak-associated genes related to biological processes of stem cell maintenance and proliferation"

Description of data in Results section 2.
TEAD1 was… "one of the only tumor-specific TFs to show differential overexpression in E+GSCs"
- Importantly, TEAD2 and TEAD3 seem to have greater overexpression in E+GSCs than TEAD1, however only TEAD1 quantification is provided in the mansucript.

"We noted widespread expression of TEAD1 but not of other TEAD members" Supp Fig2.
- A lack of signal in immunofluorescence can be due to many factors. Some form of positive staining to show the antibody works under similar conditions (for TEAD4) is needed.
- TEAD3 was not tested.
- TEAD2 and TEAD3 has RNA expression shown in Fig 2C

Figure 3B
- Not defined what the numbers in this figure represent.
- Raw data for "genes significantly coexpressed with TEAD1 in TCGA GBM samples" is not provided.

Figure 4B
- Images for migration do not have scale bars.
Invasion assays
- Invasion assays were run for 48 hours. Cell growth under the different conditions tested needs to be assessed and described. If the cell number on the bottom of the transwell dish is compared to the original cell number plated then this even more necessary.

Results section 4.
- Statements for a "negative effect on stem cell maintenance" mention limiting dilution assay and formation of spheres with smaller diameters. Sphere diameter is not typically used as a measure of stem cell maintenance; this statement be amended or clarified with references.

Fig 5 A.
- It is unclear what the size of the sections in the pie chart represent.

Fig. 6 A,B.
- Quantification of the western blot (A) are not included in the chart (B) to show the protein levels of EGFR.

Fig. 6 C.
- There is no scale for the peaks at the EGFR TSS.

3. Methods:

Method for associating ATAC-seq peaks to the nearest gene is not described.

More than one sequence for CRISPR gRNA should be used for all the knockouts. This is especially important as the TEAD1 overexpression was not sufficient to rescue EGFR expression.

Invasion assay: the growth factors and serum attractants used were not sufficiently described.

ELDA assay: more details for this experiment are required. How many wells were used? How many replicates were performed? What was the size cutoff for counting spheres?

qPCR: How many replicates were performed? The authors only mention that duplicate wells were used

Dear Reviewers,

Thank you very much for your comments on our manuscript, now entitled **"Chromatin accessibility uncovers TEAD1 as a regulator of migration in human glioblastoma"**. Overall, we found the critiques and suggestions by all three reviewers helpful and constructive, and are happy to report that after seven months of rigorous experimental studies, we have been able to address most of them, making our message stronger and clearer.

To summarize, this is a unique human study that employs epigenetics to uncover novel transcriptional drivers and targets of cell migration in human glioblastoma stem cells (GSCs), by capturing the cells' native molecular imprint and developmental context in tissue-derived (uncultured) conditions. By uniquely contrasting chromatin accessibility (via ATAC-seq) in GBM to early germinal growth, we were able to distinguish developmental and tumor-specific chromatin states within GSCs, and to define a regulatory signature centered around the TEAD transcriptional family, which relates specifically to GSC migration. Subsequent functional validation experiments using CRISPR-Cas9 ablation of TEAD1 in primary GBM cells validated a role for TEAD1 in tumor migration and began to uncover some of TEAD1's transcriptional targets, including the EGFR gene.

Following the suggestions of all three reviewers and the editor's advice, we have performed several additional studies, which further substantiate the role of TEAD1 in tumor migration, both in vitro and in vivo, and provide mechanistic insight into how TEAD1 regulates GBM migration. Important improvements in our revised manuscript include:

1) **In vivo validation of TEAD1's role in tumor migration** in xenograft models. Following R2's advice and yours, we performed xenotransplantation of GBM cells with CRISPR-CAS9-mediated ablation of TEAD1 and found that the resultant patient-derived xenografts had marked deficits in tumor infiltration.

2) **Reproducibility of TEAD1-knockout migratory deficits** in additional primary GBM cell lines. Although this was not a major concern by any of the reviewers, we felt compelled to validate our observations in three additional primary GBM cell lines (representative of all GBM subtypes), all of which displayed deficits in tumor migration in vitro. Moreover, we confirmed a direct role for TEAD1 in cell migration, by demonstrating that TEAD1 overexpression partially rescues migratory deficits in TEAD1-knockout cells. Of note, we also performed functional and transcriptome (see below) analysis of TEAD4 knockout cells across the four primary GBM cell lines but found overall less consistency in their migratory deficits and a lack of a defined transcriptome signature.

3) Generation and analysis of **TEAD1-knockout RNA-seq data across GBM types and in the context of migration**. Following R2's suggestion, we generated RNA-seq data from sham and TEAD1-knockout lines and spheroids (4 cell lines and 3 spheroid migration experiments) using which we were able to define a

clear TEAD1-transcriptome signature in the context of migration and to hone in on specific migratory TEAD1-targets conserved across GBM subtypes.

4) **Mechanistic insight into TEAD1's downstream regulators of tumor migration.** Multidimensional analysis of ATAC-seq, ChIP-PCR, and RNA-seq data uncovered previously unknown TEAD1-dependent regulators in the context of tumor migration, including AQP4 and the mesenchymal cadherins CDH4 and CDH11. Overexpression of AQP4 or CDH11 restored migratory deficits in TEAD1-knockout cells. Furthermore, we validated AQP4 as a direct binding target for TEAD1, and demonstrated that both AQP4 expression and migratory deficits are rescued after TEAD1 overexpression. Mechanistically, our study now points towards a direct TEAD1-mediated transcriptional regulation of AQP4, a previously unrecognized TEAD1-target interaction, to promote GBM cell migration.

In summary, our additional studies provide even stronger evidence for the regulatory role of TEAD1 in GBM migration, both in vitro and in vivo, and implicate for the first time AQP4 as a TEAD1-regulatory target in this context. We are confident in our results and in the great value of this study as a resource to the scientific community.

We thank you in advance for your timely consideration.

Sincerely,

Nadejda Tsankova

Reviewer #1:

Tome-Garcia et al leverage a recent publication from their team where brain stem cells from normal (germinal matrix) and glioblastoma can be studied for shared and distinct epigenetic events that underscore glioma pathology, specifically stem cell self renewal and cell dispersion/invasion. The experimental design, to compare/contrast EGF+ cells from human GM and GBM, absent cell culture propagation, is innovative and (likely) incisive. While the ideal comparison would have been cognate representation of GM and GBM from the same individual, the executed study of allogenic pairs is a best approximation, strengthened by the clustering of chromosome accessibility marks within the different GM specimens distinct from GBM cases. Delivering the unique results of ATAC Seq data on GBM stem cells and GM stem cells the manuscript is attractive.

We thank the reviewer for capturing the overall message and positive contribution of our study.

Fig 1 illustrates the cell sorting outcome for populations that do/do not bind EGF from GM and GBM fresh isolates, and the consequent comparisons of differentially accessible peaks that are proximal to genes (likely the least rigorous assignment of mechanistic association between chromosome accessible measurement and gene expression – RNAseq strengthens this), but nonetheless uncovering stem cell proliferative related (GO Term enrichment) genes when comparing EGF binding stem cells (GM and GBM) with EGF non-binding GBM cells (posited as "developmental associated" genes). The comparison of genes proximal to chromosome accessible regions in GBM CSCs (EGF binding) distinct from GM EGF binding and GBM non-EGF binding cells uncovered genes whose most statistically significant GO terms are enriched for EGFR signaling and cell invasion behaviors. It would be instructive to report how many genes were associated with 10,509 accessible peaks in E+GSC cells – will do.

We now report in the text that the 10,509 differentially accessible peaks in our GSC tumor-specific ATAC-seq analysis correspond to 4015 protein-coding genes. Of these, 2612 protein-coding genes contained at least one peak with associated TEAD1/4 motif (revised Fig. 7a).

Inferring chromosome accessible regions to transcription factor motifs using HOMER de novo motif-discovery algorithm is shown in Fig 2. 26 tumor-specific motifs were uncovered, while 27 motifs shared between GM and GBM EGF binding (stem) cells were noted. [1]Precisely how these listed TFs were prioritized is unclearly described, and the grooming to the top candidate TFs ("…exclusively enriched in only one of the [chromosome accessible] peaks…") is very unclear; likely making this discovery unreproducible by another lab. [2]The comparison in Fig 2C (between E+GSC and E-GBM seems misdirected relative to the text, "TEAD1 was the most highly expressed member of the TEAD family in these GBM cells (Fig 2c)…". The heatmap shows almost no difference in TEAD1 expression (fig 2c), and the difference in Fig 2d (~9.7 to 10.5) while being statistically different numerically, is of dubious persuasion. How does this data qualify elevation above the other TF candidates? Since gene expression from the different cell populations already exists (Ref 16), why not show the comparison of the other promising candidates as well (similar to Fig 2d)?

[1]We have clarified better our motif prioritization strategy in the text.
[2]Following the reviewer's advice, we have converted our heatmap figure into a bar graph, which depicts more clearly the absolute gene expression values for tumor-specific TF candidate genes as well as the significance in their differential expression between E+GSC and E-GBM populations (revised Fig. 2c). We describe more clearly in the results and discussion sections our specific qualifications for choosing TEAD1 above other motifs for subsequent functional validation.

[1]The described agreement between Fig 3A and Fig 2c (expression patterns of TEAD family members in TCGA

versus the E+GSC) is unpersuasive in viewing the data shown. [2]Along with the immunofluorescent images in Suppl fig 2 for TEAD1, TEAD2 and TEAD4, the declaration (in Results Section 3) that, "…this analysis prioritized TEAD1 as the most highly expressed TEAD family member in GBM, and implicated its relevance in tumor migration, warranting further functional validation in cGSCs" seems to overstate the presented data. [3]Genes co-expressed with TEAD1 in TCGA GBM samples (Spearman's correlation BH-adjusted p<0.05) being highly enriched for terms related to cell migration and cell adhesion (Fig 3B) do not reach a persuasive level warranting including of Fig 3 in the main text of the manuscript.

[1]We now represent the data in previous Figs. 3A and 2C (expression pattern of TEAD family members in TCGA and E+GCS) next to each other (revised Fig. 2d+e), which visualized more clearly the similar expression pattern seen in the two datasets.
[2]We agree with the reviewer about the overstatement in our presented data and have modified the text accordingly.
[3]We have placed previous Fig. 3b in supplementary materials (revised supplementary Fig. 2b). The finding of functional enrichment for migratory and adhesion terms in TEAD1-coexpressed genes across the TCGA dataset is correlative, but it does supplement the overall findings in our study.

Fig 4 is a compelling depiction of a role of TEAD1 in GBM migration, invasion, and self-renewal in the limited sample set studied.

We have now generated additional data that provides further support for the role of TEAD1 in cell migration. First, we confirmed that TEAD1-knockout displays cell migration deficits in three additional patient-derived GBM lines (revised supplementary Fig. 4). Second, we found that TEAD1 overexpression in TEAD1-knockout cells restores their migratory phenotype (revised Fig. 3g). Finally, we demonstrated that xenotransplantation of TEAD1-knockout cells also yields significant deficits in tumor infiltration in vivo (revised Fig. 4).

[1]Fig 5A is inadequately described, leading to confusion as to its message. [2]Fig 5B does not convey a coherent elaboration of the findings; this warrants either better integration to the evolving description of discovery, or elimination. [3]Fig 5C has 14 rows of heatmapped squares, but the assigned genes number only 13; this discrepancy should be readily remedied. [4]The election to focus solely on EGFR (p=1.89e-2) compared to ITGA7 (p=6.47e-06) or other more statistically significant edges (fig 5d) should be better explained.

[1]We provided more accurate description of the data presented in Fig. 5a.
[2]We improved the coherency of this description and moved Fig. 5b and Fig. 5c to supplementary materials (revised supplementary figs. 5a and 5b) in order to maintain the focus of Fig. 5 on the validated set of TEAD1-target genes.
[3]We regret this oversight and have now fixed the heatmap to have corresponding squares to genes (revised Supplementary Fig. 5b).
[4]We clarified in the text our choice for validating EGFR and other selected targets (see below), which we based on the consideration of our ATAC-seq data (accessibility peak number and underlying motif scores) and differential E+GSC expression, rather than on the TEAD1 TCGA gene co-expression analysis. The latter has been moved to supplementary Fig. 2b in order to avoid confusion.

Fig 6 portrays results linking TEAD1 as a TF controlling expression of EGFR. The closing statement in the Results section, "…TEAD1 as an important TF regulator of the migratory phenotype in primary GSCs, and strongly implicates EGFR as one of its downstream targets" is supported by the data, but is nonetheless overly exclusive of the other associated genes in Fig 5, which are not further developed in this manuscript.

We agree and have now extended our in vivo validation analysis to include additional genes from Fig. 5. We also expanded our ChIP-PCR analysis to include larger number of GBM samples (n=6), in which we validate TEAD1 binding in trans at *EGFR*, *CDH4*, and *AQP4*. This data is now depicted in the revised Fig. 5. We dedicate a separate figure (revised Fig. 6) to the TEAD1-dependent regulation of EGFR and downstream EGFR signaling, which we find to be associated with consistent downregulation of pERK but not pAKT.

[1]The Discussion is a careful retelling of the results, integrating them into a glioma biology framework where TEAD1 is portrayed as a TF regulating GBM invasion and TEAD4 playing a role in GBM self-renewal (stem like behavior). The other associated genes are called out as meaningful but "beyond the scope of this study". [2]The closing paragraph in the Discussion borders on overstatement, since TEAD1-associated regulation of EGFR does not particularly guide "…us a step closer to targeting the diffusely infiltrative nature of this fatal disease".

[1]Our expanded analysis provides strong validation for the role of TEAD1 in cell migration, both in vitro and in vivo, and shows somewhat redundant role of TEAD4 for cell migration in vitro. Unlike TEAD1, knockout of TEAD4 showed less consistent migratory deficits across the four cell lines examined, and lacked a defined transcriptome signature in our additional RNA-seq knockout data analyses. Therefore, our conclusions and discussion now focus primarily on TEAD1, which our data supports as the more consistent regulator of tumor migration across GBMs.
[2]In complete agreement with the reviewer, we have modified the closing paragraph to remove the overstatement and reflect better the conclusions of our study as they relate to EGFR.

The Methods section is reasonably detailed to enable reproduction of the experiments with the exception of the critique of the data in Fig 2 (above).

We have now clarified the selection critique of enriched TF motifs (Fig. 2) in the text and in the methods section.

Reviewer #2:

Tome-Garcia et al's manuscript titled "Chromatin accessibility uncovers TEAD1 as a regulator of migration and EGFR expression in human glioblastoma" wished to address the specific chromatin status and binding transcription factors (TFs) in glioma stem cells (GSCs). Using the ATAC-seq technology, they identified the regulatory GBM signatures, one of which was tumor-specific related to migration. TF motif analysis prioritized the TEAD family at tumor-specific regulatory regions: the most frequent representation at the epidermal growth factor receptor (EGFR) locus. They concluded that TEAD1 as an important TF regulator in tumor migration, and EGFR as one of the direct targets of TEAD1. This study endeavored to clarify the interaction between chromatin signature and TFs in gliomagenesis and showed some interesting data. However, the paper has significant flaws in the validity of the approach, interpretation of the data and the quality of the presentation.

Specific comments:
1. Detail of their experiments are not well explained. Although authors described that the protocol was optimized using three technical replicates for 25K cells, how consistent between each data set is? They appeared to examine multiple GBM cells and NSPCs. Are they derived from multiple patients? How many patients were examined? How consistent the ATAC-seq data between each GBM patient? These information are mandatorily required in detail to draw unbiased results.

We now provide comprehensive and more detailed explanation of our samples and experiments, which is

summarized in revised supplementary table 1. We first optimized the ATAC-seq technique in our laboratory using technical replicates of U87 cells. We are happy to provide these results, if requested. We then examined acutely isolated E+/E- GBM populations from four different GBM patients/tumors and three different autopsy GM dissection specimens. We collected only live cells (DAPI-negative for GM and DAPI-low for GBM). Analysis of primary human cells and tissues (in this case without any cell culture manipulation) always renders certain amount of variability, which we tried to minimize by focusing on patients with de-novo IDH-wildtype GBM resections and on GM dissections of similar age. The provided data in supplementary figure 1 (which now also includes TSS-centered distribution of peaks) served as quality control in our analyses, and confirmed acceptable consistency across samples.

2. [1]Since the study endeavor to show the importance of TEAD1 during gliomagenesis, genome-wide TEAD1 binding (i.e. ChIP-seq) should be shown in addition to ATAC-seq data in E+/- GBM cells, GSCs, and NSPCs. Instead, they just examined the RNA-seq and ATAC-seq datasets, which were generated from the similar samples in their previous study. They identified EGFR as a TEAD1 target using expression data and motif analysis. However, these sets of analyses may suffer from misinterpretation (vague why suffers from misinterpretation). [2]In addition, they need to perform RNA-seq expression analysis using the cells with and without TEAD1 (i.e. TEAD1 knockout cells). This study lacks these important experiments to validate their in silico analyses.

[1]Given the limited availability of fresh human tissues, and our premise to characterize TF binding in E+GSC in uncultured conditions, we were able to only obtain sufficient material of freshly isolated cells for ATAC-seq analysis (25K cells) but not for ChIP-seq analysis (would require 500K cells or more). By focusing on sorted and functionally defined populations, we obtained high-resolution ATAC-seq data for motif enrichment analysis, which allowed us to prioritize TEAD1-binding targets in cis. While we could not perform ChIP-seq, we were able to validate several of the cis TEAD1-binding targets in trans, using TEAD1 ChIP-PCR in primary GBM tissues (revised Fig. 5).

[2]Following the reviewer's suggestion, we generated RNA-seq data from sham, TEAD1-knockout, and TEAD4-knockout cells from 4 different cell lines and from three spheroid migration experiments (18 different samples overall), which provided us with robust statistical power to characterize the TEAD1-depedent transcriptome signature (see below).

3. As authors described, activity of TAZ-TEAD4 has previously been implicated in GBM cell motility and mesenchymal transformation. Although authors claimed that the specific role of TEAD1 in regulating a pro-migration GSC, they failed to show the specific and mechanistic roles of TEAD1-EGFR in gliomagenesis in the study. What is the mechanisms down-stream of TEAD1-EGFR, which affect the GBM cell features, especially stemness and migration? Which molecules are involved? These mechanistic analyses are lacking in the study.

We appreciate the reviewer's critical analysis of our study. In our revised manuscript, we provide additional data regarding the affected oncogenic targets downstream of TEAD-EGFR as well as new targets, which we now implicate in TEAD1-mediated cell migration. We assessed the expression of downstream EGFR signaling components and found that levels of pERK (but not pAKT) are significantly downregulated across four different TEAD1-knockout primary GBM cell lines. To understand the mechanism by which TEAD1 regulates migration, we first performed RNA-seq analysis in a migratory context, by examining expression within migratory sham vs. migration-deficient TEAD1KO spheroids (three technical replicates with robust consistency). Differential gene expression analysis revealed dysregulation of genes related to cell migration and EMT, rather than EGFR signaling, with consistent downregulation of AQP4 and the mesenchymal cadherin CDH11. Overexpression of either AQP4 or CDH11 restored migratory deficits in TEAD1-knockout

cells. Furthermore, we validated AQP4 as a direct binding target for TEAD1, and demonstrated that both AQP4 expression and migratory deficits are rescued after TEAD1 overexpression. Mechanistically, our study now points towards a direct TEAD1-mediated transcriptional regulation of AQP4, a previously unrecognized TEAD1-target interaction, to promote GBM cell migration.

4. Authors need to CRISPR-Cas9-mediated ablation of TEAD1 in E+/E–GBM cells.

Unfortunately, our acutely isolated E+/E- GBM model did not provide sufficient cell yield to perform the suggested CRISPR-Cas9 experiment.

5. In vivo functional analysis of TEAD1 is required. For example, conditional CRISPR-Cas9-mediated ablation of TEAD1 in orthotopic xenotransplantation model may help understanding the TEAD1 function in vivo.

The reviewer raised an important point, which we have addressed. As suggested, we generated an orthotopic xenotransplantation mouse glioma model with CRISPR-Cas9 Sham vs. TEAD1-ablated glioma cells and characterized their migratory behavior in vivo. We found that TEAD1-knockout cells display robust deficits for tumor infiltration in vivo, recapitulating our in vitro findings, which underscored further the role of TEAD1 in GBM migration.

Reviewer #3:

In this manuscript, the authors used Chromatin accessibility mapping via ATAC-seq to define two regulatory GBM signatures—a developmentally shared one related to stem cell proliferation, and a tumor-specific one related to migration. They further focused on the migratory signature and transcription factor (TF) motif discovery and the TEAD family at tumor-specific regulatory regions. To further validate the role of TEAD1 in GBM migration and further explore its relationship to EGFR, they generated TEAD1, TEAD4, and EGFR knockouts in patient-derived cultured GBM stem-like cells (CGSCs) using CRISPR-Cas9 technology. The authors conclude that eliminating TEAD1 or TEAD4 diminished migratory properties in CGSCs, mimicking the EGFR knockout effect, and repressed several putative TEAD-targets, including EGFR. They further suggested that chromatin immunoprecipitation data confirmed the specific binding of TEAD1 at an upstream regulatory locus of EGFR.

Overall comments:

This study does not convincingly show that TEAD1 is a key regulator of the invasive nature of GBM or [2]that it is a direct regulator of EGFR expression.

Major issues:

This paper highlights the infiltrative nature of GBM as a primary cause for its poor prognosis and concludes that there is a major role of TEAD1 in this process, as well as a role for TEAD1 in regulating EGFR expression.

1. However, this is not convincingly shown in the data presented in the manuscript:

[1] - Fig 4C shows that TEAD1 does not have a significant effect on GSC invasion.

[1]In the original version of this paper, all transwell invasion assays were performed at 48 hours. Following the reviewer's comment on the possible effect of proliferation at this time point, we performed proliferation assay on our cell lines, which showed a TEAD1-depenent effect on proliferation at 48hrs.

Therefore, we repeated our transwell invasion assays at 24 hours, a more commonly used time point for this assay. In our repeat experiments, we see significant decrease in cell invasion in both TEAD1 and TEAD4 knockout cells. To ensure reproducibility of our results, we generated TEAD1 and TEAD4 CRISPR-Cas9 knockout in three additional patient-derived cell lines (of different molecular subtype) and assessed cell invasion at 24 hours using the transwell assay. In all cell lines, we observed significant migratory deficits in TEAD1-knockout cells and somewhat more variable results in TEAD4-knockout cells. Furthermore, we also transplanted TEAD1-knockout cells into immunocompromised mice to generate patient-derived xenograft gliomas (suggested by another reviewer), in which we confirmed that TEAD1-knockout cells also display significant deficits for tumor infiltration in vivo (revised Fig. 4). Finally, we performed extensive RNA-seq analysis in our knockout cell lines (as suggested by another reviewer), which demonstrated significant dysregulation of the migratory and EMT gene sets in our TEAD1 (but not TEAD4) knockout cells across four primary GBM lines and in the context of migration. These results strongly implicate a role for TEAD1 in GBM cell migration, both in vitro and in vivo.

[2] - No differences in TEAD1 expression were seen in tumor core compared to areas of infiltration in Supp. Fig 2. The authors state "we did not observe significantly higher protein expression at the infiltrative tumor element"

[2]Similarly to other transcriptional regulators, we believe that TEAD1's activity on tumor migration is modulated through its differential binding at pro-migratory gene targets rather than through differential expression in migratory cells. Our data points towards several such targets, including AQP4 and CDH4.

[3] - No significant binding of TEAD1 over IgG control in ChIP-PCR to the promoter of EGFR was demonstrated

[3]We agree that our original ChIP-PCR data (n=3) was limited. We have now repeated our ChIP-PCR analysis on additional GBM tumors (n=6), which shows significant enrichment of TEAD1 (but not TEAD4) at the EGFR distant promoter above IgG. In addition, we validated TEAD1 binding in trans at additional targets, including CDH4 and AQP4, which similarly show significant TEAD1 occupancy above IgG at previously defined cis regulatory elements.

2. It is often unclear exactly which samples were being used for various experiments. Multiple statements throughout the manuscript describe the use of "similar samples" or "similar cells". It would have been useful to understand which samples were used for experiments in order to allow for critical assessment and interpretation of the data presented in the manuscript.

We apologize for the confusion. We now provide a comprehensive summary table, which lists all primary samples used, their origin and method of derivation, and the exact experiment for which they were used.

3. Cell growth was not addressed in any of the experimental data presented throughout the paper. This is surprising considering the well-described role of EGFR on cell proliferation in GBM.

In agreement, we now provide cell proliferation curves for all cell lines used in our in vitro experiments, and assess proliferation in vivo using Ki67 immunohistochemistry (revised Fig. 3b, Fig. 4c). Please also see response below regarding minor comments in Figure 4B.

4. [1]*The authors describe the use of EGF ligand conjugated to APC for flow sorting for the E+GSC samples. Mutational status of the lines used in experiments (relevant to point 2 above) was not provided. [2]The reviewer also questions how an EGFRvIII sample can be sorted using the EGF-APC condition since EGFRvIII does not bind the EGF ligand.*

[1]We now provide the mutational status for all samples and cell lines used in our experiments (summarized in revised supplementary table 1).

[2]The question of whether EGF ligand can isolate tumor cells with truncated EGFR ectodomain (such as EGFRvIII mutant cells) is an important one. It has been addressed in our previous publication (Tome-Garcia et al. Stem Cell Rep 2017). Although E+GSCs were enriched for EGFR with intact ligand-binding domain, EGFRvIII could be detected in both E+ and E- fractions (likely because many tumor cells show heterogeneous EGFR wildtype / vIII mutant expression), and it did not have a direct effect on stem cell properties in our tested tumors.

Minor issues:

1. Intro Paragraph 2
"Epigenetic plasticity is critical in maintaining the stem-cell state during normal development…"
[1] - This statement should be clarifies and a reference specifically describes this phenomenon provided.
[2] - The description of "tumor-specific" analysis is not clear as the analysis compares GSCs to GBM and NSPCs, when comparing to GBM it is therefore not tumor-specific.

[1]The statement has been now clarified and appropriately referenced in the text.
[2]This is a great point. We have now added "GSC tumor-specific" rather than simply "tumor-specific".

2. Results Paragraph 1.

"GBM tumors differ substantially from neural progenitors in their malignant spread and progression."
- Statement is not clear.

We have clarified this statement in the text.

Missing reference in Results section 1.
- After "Consistent with previous reports, the developmentally shared GSC/NSPC peak-associated genes related to biological processes of stem cell maintenance and proliferation"

We have added a reference after the statement.

Description of data in Results section 2.
TEAD1 was… "one of the only tumor-specific TFs to show differential overexpression in E+GSCs"
- Importantly, TEAD2 and TEAD3 seem to have greater overexpression in E+GSCs than TEAD1, however only TEAD1 quantification is provided in the manuscript.

Our previous heatmap depiction of TEAD1-4 RNA-seq expression data (previous Figure 2C) did not reflect on the significance of E+GSC overexpression of TF genes. Following critique by two reviewers, we have now replaced the heatmap with a bar graph representation, in which we include absolute rld-normalized gene expression values and significant E+GSC enrichment for top-enriched TF candidates from our Homer de novo analysis (revised Fig. 2c). We include TEAD1 and TEAD4 motifs because these were the top-enriched motifs derived from the Homer analysis. TEAD2 (but not TEAD3) showed significant enrichment in E+GSCs but we did not pursue this TEAD family member further because its absolute expression was lower than TEAD1 in the E+GSC and TCGA samples (which we now depict using bar graphs in revised Fig. 2d-e), and because we did not detect TEAD2 protein expression in our in vivo PDX model system. For similar reasons,

we did not pursue further TEAD3. We analyzed TEAD4 in addition to TEAD1 because its motif was enriched and because most previous GBM studies had focused on TEAD4; thus we thought its analysis can serve as a positive control. This justification is now delineated more clearly in the results section.

"We noted widespread expression of TEAD1 but not of other TEAD members" Supp Fig2.
[1] - A lack of signal in immunofluorescence can be due to many factors. Some form of positive staining to show the antibody works under similar conditions (for TEAD4) is needed.

[1]We absolutely agree with this comment and now include positive control staining for TEAD4, derived from our in vitro cell lines (Supplementary Fig. 3c). Interestingly, expression of TEAD4 was more prominent in vitro than in vivo.

[2] - TEAD3 was not tested.
[3] - TEAD2 and TEAD3 has RNA expression shown in Fig 2C

[2-3]In our analysis of TCGA RNA-seq data, we found that expression of *TEAD2* and *TEAD3* is much lower than of *TEAD1* (2.5 and 4 log_2 scale lower, amounting to 6.25 and 16 times lower absolute reads, respectively). Since we did not detect significant protein expression of TEAD2 in vivo, we did not think it is worth pursuing the even lower expressor TEAD3.

Figure 3B
[1] - Not defined what the numbers in this figure represent.
[2] - Raw data for "genes significantly coexpressed with TEAD1 in TCGA GBM samples" is not provided.

[1]The numbers in this figure represent fold enrichment, which we have documented now in the figure legend (supplementary Fig. 2c). [2]We have also included the requested raw data for "genes significantly coexpressed with TEAD1 in TCGA GBM samples" as Supplementary Data 2.

Figure 4B
[1] - Images for migration do not have scale bars.
Invasion assays
[2] - Invasion assays were run for 48 hours. Cell growth under the different conditions tested needs to be assessed and described. If the cell number on the bottom of the transwell dish is compared to the original cell number plated then this even more necessary.

[1]The original spheroid migration images had very small scale bars, which we have now magnified so that they are readily seen.

[2]This was an important critique, which we have now thoroughly addressed. Cell growth assays revealed proliferation 24-36 hours after attachment. Given this, we repeated all of our transwell invasion experiments at 24 hours (previously they were done at 48 hours). Please see our above responses on detailed description of our new results. We also quantitate cell growth in our in vivo functional analysis using Ki67 immunofluorescence.

Results section 4.
- Statements for a "negative effect on stem cell maintenance" mention limiting dilution assay and formation of spheres with smaller diameters. Sphere diameter is not typically used as a measure of stem cell maintenance; this statement be amended or clarified with references.

We appreciate the reviewer's expert comment. We now describe our findings on sphere diameter independent of stem cell maintenance.

Fig 5 A.
- It is unclear what the size of the sections in the pie chart represent.

We have clarified in the figure legend (revised Supplementary Fig. 5a) that the size of the sections in the pie chart represents "Percent of gene hit against total number of pathway hits".

Fig. 6 A,B.
- Quantification of the western blot (A) are not included in the chart (B) to show the protein levels of EGFR.

We believe the reviewer is referring to the lack of TEAD protein quantification (rather than EGFR) in this figure, which we now appropriately provide (revised Fig. 6b).

Fig. 6 C.
- There is no scale for the peaks at the EGFR TSS.

We apologize for the oversight and now provide the scales for EGFR TSS and IN2 IGV peak tracks (revised Fig. 5b).

3. Methods:

[1]Method for associating ATAC-seq peaks to the nearest gene is not described.
[2]More than one sequence for CRISPR gRNA should be used for all the knockouts. This is especially important as the TEAD1 overexpression was not sufficient to rescue EGFR expression. (TRIED BUT DID NOT DO EVENTUALLY Tead1a and TEAD1b but did we ever combine? We tried combining a bunch and got worse results, in our hands – not full KO or redundancy – show all the failed KO experiments Jessica tried in the last 3 months)
[3]Invasion assay: the growth factors and serum attractants used were not sufficiently described.
[4]ELDA assay: more details for this experiment are required. How many wells were used? How many replicates were performed? What was the size cutoff for counting spheres?
[5]qPCR: How many replicates were performed? The authors only mention that duplicate wells were used.

We now provide further detail in the methods section on [1]the exact parameters used to associate peaks to genes using both the GREAT and HOMER tools, [3]the growth factors and serum attractants used in our cell invasion assay, and the [4]ELDA assay number of wells, replicates, and sphere size cut off. [2]Depending on the specific CRISPR-Cas9 experiment, we used one or two gRNAs per gene. If one gRNA was sufficient to cause the desired mutation and eliminate more than 95% of protein expression, we did not infect with a second gRNA. [5]We have substituted the qPCR data with deep sequencing RNA-seq data, performed in four independent biological replicates.

Reviewer #1 (Remarks to the Author):

Tome-Garcia et al have revised the manuscript in accord with the critique of the prior draft, including revision of figures, expanded text, shuffling figures to supplemental status, etc. Additional patient-derived GSCs have been included in the current version, confirming the discovery and validation of transcription factor motifs from ATAC Seq in uncovering candidate driving events of glioblastoma invasion and migration.

Laudable contributions conveyed by the report derive from the use of non-cultured, freshly-collected human E+NPSC (at autopsy) against which to compare E+GSC (from tumor resection) as the input material in the ATAC Seq discovery exercise.

Inadequately described informatic methods for grooming the chromatin accessibility measurements to uncover tumor-specific (distinct from developmental-specific) open chromatin regions which were subsequently mapped to genes sets and GO Term Enrichment have been greatly improved. More granular explanation of genes that are tumor-specific strengthens the manuscript. The process and rational by which Tumor-Specific analysis of genes (HOMER de novo motif discovery) are now robustly described, making Figure 2 a more clear maturation of the discovery steps to get to TEAD4/1 as a lead TF candidate underlying glioma invasion. Abundance of individual TFs may not be the sole driving determinant of biological significance in gene regulation, since multiple TFs, as well as cofactors, influence gene expression; pre-mRNA processing, transit to cytoplasm, guidance to ribosomal machinery for translation, and numerous post-translational modifications all impact functional biology, which the manuscript should acknowledge at even a cursory level.

Fig 4 remains a compelling depiction of a role of TEAD1 in GBM migration, invasion, and self renewal in the sample set studied.

Figs 5 and 6 are much improved by use of bar charts rendering ChIP-Seq data advancing TEAD1 over TEAD4 as the mechanistic driver of specific regulation of EGFR, AQP4, and CDH4 (fig 5), and the western blots (Fig 6).

There is confusion by the (unanticipated and somewhat unjustified) elevation of CDH11 as a mediator of TEAD1-driven glioma invasion (Fig 7) and Discussion. The data indicate that CDH11 is NOT a TEAD1-dependent gene. The Discussion states: “Nevertheless, we did observe consistent downregulation of several TEAD1-dependent genes across cell lines and migratory experiments, including CDH11 and AQP4; the latter was also validated as a TEAD1-binding target in vivo.” And then shortly after, “While CDH11 overexpression also reversed partially migratory deficits in TEAD1-

knockout spheroids, its expression was not directly regulated by TEAD1 and we did not detect TEAD1 occupancy in any chromatin accessible regions associated with CDH11." The portion of the findings addressing CDH11 is very confusing, detracting considerably from an otherwise somewhat clean (if narrowly focused) reporting on a role of AQP4 on glioma stem cell invasion.

minor points:

p. 4. "…Tn5 transposase to bind and tagment accessible nucleosome…." What is tagment?

p.5. "…interestingly, TEAD1 KO spheres were significantly lower in size…" Replace "lower" with "smaller".

p.7. "…downregulation of pERK (Fig 6c) but not pAKT (Fig 6d)". There is no Fig 6d; this should be Fig 6c

p.23. Figure 4 legend. "…the first and the last serial section examined." Might be better rendered: "…the first and the last serial section containing tumor cells."

p.24. Figure 6 legend. "…knockout xenografts after 3.5 months." Might be better rendered: "…knockout xenografts of G-13063 cells after 3.5 months."

p.24. Figure 7 legend. Specify what the Bold and the red text represent distinct from non-bold, black text.

Reviewer #2 (Remarks to the Author):

Overall, the authors have almost addressed the concerns by performing additional experiments and supplying data. The paper has been improved. However, unfortunately, #3 and #4 mechanistic questions were not well proven by the authors. Spheroid migration assay presented in Fig. 7 is not enough to clarify the effects of AQP4 and CDH11on glioma stemness and ability for migration. Especially, it cannot reflect the physiological condition in brain tissues. How can AQP4 and CDH11 affect the glioma stemness and ability for migration? What is the mechanism?

Reviewer #3 (Remarks to the Author):

The revisions have been completed to the reviewer's satisfaction. No further changes required.

Icahn School of Medicine at Mount Sinai

Nadejda Tsankova, MD/PhD
Associate Professor
Department of Pathology & Neuroscience, Friedman Brain Institute
Icahn School of Medicine at Mount Sinai
1425 Madison Ave: Icahn 9-20E; New York, NY 10029
Nadejda.tsankova@mssm.edu

July 27th, 2018

Dear Dr. Carolina Perdigoto and team,

Please find below our itemized response to the additional comments raised by the reviewers during this second round of revisions of our manuscript now entitled "**Analysis of chromatin accessibility uncovers TEAD1 as a regulator of migration in human glioblastoma"**. We appreciate the additional comments and critiques, which we found helpful in further improving our final manuscript by making our overall message more accurate and clear.

Reviewer #1 (Remarks to the Author):

Tome-Garcia et al have revised the manuscript in accord with the critique of the prior draft, including revision of figures, expanded text, shuffling figures to supplemental status, etc. Additional patient-derived GSCs have been included in the current version, confirming the discovery and validation of transcription factor motifs from ATAC Seq in uncovering candidate driving events of glioblastoma invasion and migration.

Laudable contributions conveyed by the report derive from the use of non-cultured, freshly-collected human E+NPSC (at autopsy) against which to compare E+GSC (from tumor resection) as the input material in the ATAC Seq discovery exercise.

Inadequately described informatic methods for grooming the chromatin accessibility measurements to uncover tumor-specific (distinct from developmental-specific) open chromatin regions which were subsequently mapped to genes sets and GO Term Enrichment have been greatly improved. More granular explanation of genes that are tumor-specific strengthens the manuscript. The process and rational by which Tumor-Specific analysis of genes (HOMER de novo motif discovery) are now robustly described, making Figure 2 a more clear maturation of the discovery steps to get to TEAD4/1 as a lead TF candidate underlying glioma invasion. Abundance of individual TFs may not be the sole driving determinant of biological significance in gene regulation, since multiple TFs, as well as cofactors, influence gene expression; pre-mRNA processing, transit to cytoplasm, guidance to ribosomal machinery for translation, and numerous post-translational modifications all impact functional biology, which the manuscript should acknowledge at even a cursory level.

We have included a short statement within the 5th paragraph of our discussion, acknowledging the complex regulatory network of tumor migration biology which undoubtedly extends beyond TEAD1, and discuss this in the context of the lack of direct regulatory relationship between TEAD1 and CDH11, even though both demonstrated pro-migratory properties in our in vitro assays.

Fig 4 remains a compelling depiction of a role of TEAD1 in GBM migration, invasion, and self renewal in the sample set studied.

Figs 5 and 6 are much improved by use of bar charts rendering ChIP-Seq data advancing TEAD1 over TEAD4

as the mechanistic driver of specific regulation of EGFR, AQP4, and CDH4 (fig 5), and the western blots (Fig 6).

There is confusion by the (unanticipated and somewhat unjustified) elevation of CDH11 as a mediator of TEAD1-driven glioma invasion (Fig 7) and Discussion. The data indicate that CDH11 is NOT a TEAD1-dependent gene. The Discussion states: "Nevertheless, we did observe consistent downregulation of several TEAD1-dependent genes across cell lines and migratory experiments, including CDH11 and AQP4; the latter was also validated as a TEAD1-binding target in vivo." And then shortly after, "While CDH11 overexpression also reversed partially migratory deficits in TEAD1-knockout spheroids, its expression was not directly regulated by TEAD1 and we did not detect TEAD1 occupancy in any chromatin accessible regions associated with CDH11." The portion of the findings addressing CDH11 is very confusing, detracting considerably from an otherwise somewhat clean (if narrowly focused) reporting on a role of AQP4 on glioma stem cell invasion.

We agree that our description of the relationship between TEAD1 and CDH11 was confusing and our word choice – not entirely accurate. We have added / modified and removed text from both the results and the discussion sections of the manuscript, clarifying that CDH11 is not a direct TEAD1 binding target in primary GBM samples and that its regulation of migration is not directly dependent on TEAD1. Throughout the manuscript (from abstract to discussion) we clarify better that our study uncovers AQP4 as the only direct TEAD1 binding target implicated in GBM migration, solidifying our focus on the direct relationship between TEAD1 and AQP4.

minor points:
p. 4. "…Tn5 transposase to bind and tagment accessible nucleosome…." What is tagment?

Tagment describes the simultaneous ability of the Tn5 enzyme to tag and fragment open chromatin.

p.5. "…interestingly, TEAD1 KO spheres were significantly lower in size…" Replace "lower" with "smaller".

Thank you, we have made the suggested replacement.

p.7. "…downregulation of pERK (Fig 6c) but not pAKT (Fig 6d)". There is no Fig 6d; this should be Fig 6c

We apologize for the oversight, we have corrected this.

p.23. Figure 4 legend. "…the first and the last serial section examined." Might be better rendered: "…the first and the last serial section containing tumor cells."

We kept the original wording in this situation because in few cases (particularly in TEAD1KO slices) no tumor cells were seen in distant sections examined. The analysis of tumor volume spread was performed using an averaged Area migration multiplied by a constant distance from section 1 to section 11, which represents the typical tumor spread we observed within sham tumors. The use of constant distance allowed us to avert underestimation of tumor spread due to sampling error.

p.24. Figure 6 legend. "…knockout xenografts after 3.5 months." Might be better rendered: "…knockout xenografts of G-13063 cells after 3.5 months."

Thank you, we have made the suggested replacement.

p.24. Figure 7 legend. Specify what the Bold and the red text represent distinct from non-bold, black text.
We removed the bold marks as they were inadvertently left from a draft version. We kept AQP4 in red to highlight that it was found to be a direct binding target of TEAD1 in trans.

Reviewer #2 (Remarks to the Author):

Overall, the authors have almost addressed the concerns by performing additional experiments and supplying data. The paper has been improved. However, unfortunately, #3 and #4 mechanistic questions were not well proven by the authors. Spheroid migration assay presented in Fig. 7 is not enough to clarify the effects of AQP4 and CDH11on glioma stemness and ability for migration. Especially, it cannot reflect the physiological condition in brain tissues. How can AQP4 and CDH11 affect the glioma stemness and ability for migration? What is the mechanism?

As per the editor's suggestions, we have addressed textually and with further discussion our findings. We have clarified in the text that the direct role of TEAD1-AQP4 in regulating GBM migration is demonstrated in the spheroid migration assay but not in vivo, toning down our overall conclusions. We hope to investigate the downstream mechanisms of TEAD1-AQP4 driven migration under physiological conditions in future studies, which we believe is an important question but beyond the scope of this study.

Reviewer #3 (Remarks to the Author):

The revisions have been completed to the reviewer's satisfaction. No further changes required.

We did not make any additional changes.

We thank you for your time and hope these revisions meet your expectations.

Sincerely,

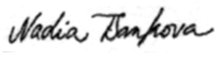

Nadejda Tsankova
